# Population fraction of Parkinson’s disease attributable to preventable risk factors

**DOI:** 10.1038/s41531-023-00603-z

**Published:** 2023-12-05

**Authors:** Haydeh Payami, Gwendolyn Cohen, Charles F. Murchison, Timothy R. Sampson, David G. Standaert, Zachary D. Wallen

**Affiliations:** 1 Department of Neurology, University of Alabama at Birmingham, Birmingham, AL 35233 USA; 2 Aligning Science Across Parkinson’s (ASAP) Collaborative Research Network, Chevy Chase, MD 20815 USA; 3 Department of Biostatistics, University of Alabama at Birmingham, Birmingham, AL 35233 USA; 4 Department of Cell Biology, Emory University School of Medicine, Atlanta, GA 30329 USA

**Keywords:** Risk factors, Parkinson's disease

## Abstract

Parkinson’s disease is the fastest-growing neurologic disease with seemingly no means of prevention. Intrinsic risk factors (age, sex, and genetics) are inescapable, but environmental factors are not. We identified repeated blows to the head in sports/combat as a potential new risk factor. 23% of PD cases in females were attributable to pesticide/herbicide exposure, and 30% of PD in males were attributable to pesticides/herbicides, military-related chemical exposures, and repeated blows to the head, and therefore could have potentially been prevented.

## Introduction

Currently, the most compelling weapon against the global rise in PD^1^ is the elimination of risk factors^2^. Studies to date have examined PD risk factors using relative risk or odds ratio (OR), which estimate the strength of association, but do not reflect their impact on disease burden. For example, as we will show here, exposure to military-related chemicals is a stronger risk factor than exposure to commonly used pesticides/herbicides by twofold, but its impact on disease burden is a third of pesticides/herbicides because one is rare, and the other is common. Here, we calculate the population attributable fraction (PAF): the percentage of disease that would be prevented if a risk factor were eliminated^3^. PAF is used to evaluate the relative impact of risk factors on disease burden to strategize intervention^4^. To our knowledge, PAF has not been applied to PD before.

The study setting was the Deep South of the United States, which is understudied, and little known about the risk factors that are operative. To that end, we began by examining the common features of PD (Table 1) to determine if data are comparable to other PD populations. Then we examined the risk factors to determine which are operative here (Table 2a, b). Having established this foundation, we calculated PAF (Table 2c). Individual-level data and the exact questions that were asked to solicit the data are in Supplementary Material.

**Table 1:** Enrollment, subject characteristics, and features of PD.

	PD		NHC		PD features	
Subject characteristics	*N* with data	Summary statistics	*N* with data	Summary statistics	OR [95% CI]	*P*
Sample size	808	–	415	–	–	–
Sex (*N* & % male)	808	512 (63%)	415	125 (30%)	–	–
PD age at onset	808	59.3 ± 11.0	–	–	–	–
Jewish ancestry	597	16 (3%)	313	9 (3%)	–	–
Black or African American	804	20 (2%)	410	6 (1%)	–	–
Hispanic or Latino	787	13 (2%)	403	3 (0·7%)	–	–
White	804	772 (96%)	410	398 (97%)	–	–
Spouses/singletons	808	116/692	415	116/299	–	–
Non-Hispanic White Spouses/Singleton^a^	763	106/657	397	106/291	–	–
Residents of the Deep South	799	764 (96%)	403	376 (93%)	–	–
Residents of DS Periphery	799	32 (4%)	403	26 (6%)	–	–
*PD features*						
Constipation	775	345 (45%)	402	57 (14%)	4.8 [3.5–6.7]	4E−25
RBD	479	47 (10%)	229	0 (0%)	50.4 [3.1–821.5]	2E−6
Weight loss	790	212 (27%)	406	56 (14%)	2.3 [1.7–3.1]	4E−7

Characteristics are shown with the number of individuals for whom specified data were available and analyzed (N with data). Summary statistics is the mean and standard deviation for quantitative traits and the number of individuals and % of the total who are positive for the characteristic of dichotomous traits. Deep South (DS) includes the states of Alabama, Mississippi, Georgia, South Carolina, and Louisiana. DS periphery states are Texas, Tennessee, North Carolina, and Florida. A question on REM sleep behavior disorder (RBD) was added at the start of the second wave of data collection and was not asked of 316 PD and 181 nurologically heathy controls (NHC) of the first wave. To assess the robustness of data, features that are known to be more prevalent in PD were tested. The preponderance of male sex among PD was tested as a proportion of persons with PD who were male (63%) vs. expected for the null hypothesis of no sex difference (50%), using the Z test (one proportion test) and showing the significance (P). Constipation, RBD, and weight loss were tested by comparing PD to NHC, using odds ratio (OR), 95% confidence interval (CI) of OR, and significance (P) denoting the difference between PD and NHC.

^a^Study was repeated for non-Hispanic Whites (to remove admixture of race and ethnicity) separated into non-overlapping groups of spousal pairs and singletons. Results were qualitatively the same (see Supplementary material).

**Table 2:** Risk factors and population attributable fraction.

		Summary statistic		(a) Univariable analysis testing each variable individually				(b) Multivariable analysis testing each variable adjusted on others						(c) Population attributable fraction for modifiable risks		
		PD	NHC	*N* with data		PD vs. NHC		*N* with data		PD vs. NHC				Determinants of PAF		PAF (95% CI)
				PD	NHC	OR [95% CI LB]	P	PD	NHC		Adj OR [95%CI LB]	P		Adj OR	Prev. in PD	
Males	Age	68.8 ± 8·6	68.2 ± 9·2	512	125	1.01 [0.99]	0.22	297	88	1.02 [0.99]			0.10			
	Family history of PD	145 (30%)	11 (9%)	480	121	4.33 [2.51]	5E−06			4.84 [2.51]			4E−05			
	MTBI/concussion	112 (23%)	20 (16%)	493	124	1.53 [0.99]	0.06			1.20 [0.68]			0.30			
	MTBI/concussion >10 y prior to PD onset	97 (20%)	20 (16%)	493	124	1.27 [0.82]	0.18			NT			NT			
	Repeated blows to the head	76 (21%)	10 (11%)	356	95	2.31 [1.28]	0.01			2.00 [1.04]			0.04	2.00	20%	10% [0.3–20%]
	Pesticide/herbicide exposure	112 (30%)	15 (15%)	375	100	2.41 [1.47]	2E−03			2.52 [1.37]			6E−03	2.52	28%	17% [7–27%]
	Pesticide/herbicide exposure duration, y	5.2 ± 13.7	3.1 ± 10·9	275	65	1.01 [0·99]	0·13			NT			NT			
	Military-related chemical exposures	27 (7%)	2 (2%)	373	99	3.78 [1.12]	0.04			6.03 [1.08]			0.04	6.03	7%	6% [0.03–12%]
Females	Age	67.5 ± 8.9	65.3 ± 8.3	296	290	1.03 [1.01]	1E−03	193	206	1.03 [1.01]			8E−03			
	Family history of PD	100 (37%)	48 (17%)	271	282	2.85 [2.04]	1E−07			2.62 [1.75]			4E−05			
	MTBI/concussion	45 (16%)	25 (9%)	284	286	1.97 [1.27]	5E−03			1.35 [0.79]			0·18			
	MTBI/concussion >10 y prior to PD onset	37 (13%)	25 (9%)	284	286	1.56 [1.00]	0.05			NT			NT			
	Repeated blows to head	0 (0%)	0 (0%)	215	195	NT	NT			NT			NT			
	Pesticide/herbicide exposure	76 (35%)	30 (14%)	219	214	3.26 [2.19]	6E−07			2·85 [1·87]			2E−05	2.85	35%	23% [12–34%]
	Pesticide/herbicide exposure duration, y	5.9 ± 13.3	2·8 ± 10·5	161	159	1.02 [1.01]	0.02			NT			NT			
	Military-related chemical exposures	1 (0.4%)	0 (0%)	224	215	NT	NT			NT			NT			

Each risk factor was tested for association with PD individually (a), those that were significant were carried into multivariate analysis (b), followed by PAF calculation for modifiable factors (c). NHC: neurologically healthy control. Summary statistics: mean ± standard deviation, or number and percentage of subjects positive for risk factor. OR: odds ratio. Adj OR: OR of each risk factor adjusted for all other variables listed. *CI* confidence interval, *LB* lower bound (upper bound is infinity), *Prev* prevalence, *y* years, *PAF* population attributable fraction, i.e., percentage of PD cases attributed to the risk factor. Results for non-Hispanic Whites spousal pairs and singletons can be found in Supplementary Material.

The study included 808 persons with PD (PwP) and 415 neurologically healthy controls (NHC). All known features of PD that were tested were captured in this population (Table 1). Notably, among PwP, 63% were males; and compared to NHC, PwP had a higher prevalence of constipation, weight loss, and rapid eye movement sleep behavior disorder (RBD). Recapitulating these features establishes the robustness of the data.

Next, we tested known risk factors to determine which are operative in the Deep South (Table 2a, b). Family history of PD and pesticide/herbicide exposure were associated with increased risk of PD in both sexes. Mild/moderate traumatic brain injury (MTBI) that results in concussion or hospitalization is reportedly more common in PD, but it may be due to reverse causality (balance problems in prodromal PD causing falls and head injury)^2^. Here, MTBI/concussion was marginally significant but not when censored to 10 years prior to the onset of PD, and the signal was lost when adjusted for other risk factors. We tested repeated blows to the head in sports or combat as a potential new risk factor and found it to be associated with PD in men. No female reported blows to the head. Exposure to military-related chemicals (i.e., chemical warfare or heavy use of herbicides (Agent Orange)) was a significant risk factor in males. Only one female, who had PD, reported exposure to military-related chemicals.

Having established the characteristic features of the study population, and the strength of association of each risk factor with PD, we then calculated PAF (Table 2c). PAF is meaningful only if intervention is feasible, hence, we calculated PAF for risk factors that are modifiable. In calculating PAF, we used OR that were adjusted on all risk factors (including age, family history, MTBI/concussion, pesticide/herbicide exposure, military-related chemical exposures, and repeated blows to the head, as applicable). Hence, PAF estimates are adjusted PAFs. Adjusted PAF for pesticide/herbicide exposure was 23% [12–33%] for females and 17% [7–27%] for males. In addition, among males, adjusted PAF was 10% [0.3–20%] for repeated blows to the head, and 6% [0.03–12%] for military-related chemical exposures. To assess the fraction of disease in males that was attributed to all three risk factors, we calculated joint PAF which accounts for potential overlap (a farmer exposed to toxicants could also have played a collision sport and had repeated blows to the head). Pesticide/herbicide, military-related chemicals, and repeated blows to the head accounted for 30% [7–49%] of PD in men.

In summary, we report two original findings: (1) Repeated blows to the head, which are common in collision sports and do not normally require medical care, were associated with a twofold increased risk of PD. (2) One in three cases of PD in males, and one in four cases in females were attributed to modifiable risk factors and could have potentially been prevented. Results were consistent across non-Hispanic White spousal pairs and singletons (Supplementary Material). Other races/ethnicities were too small for stratified analyses.

The association of PD with repeated blows to the head that do not have a medical manifestation was a notable finding in this study and should be tested and confirmed in other studies. The most common source of repeated blows to the head is collision sports. Studies have shown adverse consequences of collision sports (e.g., football) on brain health later in life^5^. It is well-publicized in the popular media that football players go on to suffer high rates of depression, mood and behavioral problems, cognitive loss, dementia, and chronic traumatic encephalopathy. Yet, the popularity of collision sports is not waning among boys and is rising among school-age girls. It is therefore imperative to implement effective safety measures for collision sports.

There are several caveats to the assessment of herbicide/pesticide exposure. Recall bias could be a confounder. Our calculations were based on self-reported exposures to heavy uses of pesticides/herbicides and did not include exposure through contaminated soil, water, fruits, vegetables, meat, and fish. Our study does not identify any one particular chemical that is directly associated with PD. In the US, few states document the use of pesticides and herbicides, with very few making such data publicly accessible. Thus, outside those areas, it is difficult to pinpoint the risk effect of one specific chemical. Mandatory and enforced registration of pesticide application would be a first step to better understand exactly which chemicals we are exposed to, at what dose, and for how long.

Some pesticides and herbicides are inherently neurotoxic. The United States allows the use of 72 pesticides that are banned in EU^6^. We still depend on certain pesticides and herbicides for modern agriculture and human health. Pyrethroids, for example, are important tools for controlling mosquito populations for limiting malaria, West Nile, and Dengue viruses. However, for certain chemicals with robust evidence of long-term harm, such as paraquat that has been firmly linked to PD, complete bans, as done in other countries, should seriously be considered.

PAF estimates derived here are not generalizable because PAF will vary across populations depending on the type and the prevalence of the risk factors in each region (e.g., rural/urban, agricultural/industrial, diet and lifestyle). There is probably variation in genetic susceptibility or past exposures and lifestyles that make people more or less vulnerable to potential damage caused by toxicant exposure or head injury. The PAF estimates provided here are a global measure that averages across various backgrounds. Deciphering interactions (which would require 4–16 times larger sample sizes) could further narrow down a subset of individuals who stand to benefit the most from intervention.

The PAF estimates reported here were based on a population of older adults and their past experiences that may have put them at risk. They are not necessarily predictive of the future. In this cohort of older Southerners, no female reported repeated blows to the head due to sports or military, and only one female (with PD) had been exposed to military-related chemicals. In the 1900’s when these women were young, women did not partake in collision sports or military, as they do now. The PAFs for the future will change, for the better or worse, depending on the actions we take now to clean our environment and improve health and safety standards.

## Methods

We have complied with all relevant ethical regulations. The study was approved by the Institutional Review Board (IRB) for the Protection of Human Subjects at the University of Alabama at Birmingham (UAB) and by the Office of Human Research Oversight (OHRO) of the United States Department of Defense (DoD, funding agency). All subjects signed informed consent. No compensation was provided for participating in the study.

We enrolled 981 persons with PD (PwP) and 485 neurologically healthy controls (NHC). Inclusion criteria for PD were diagnosis of PD by a movement disorder specialist neurologist at UAB according to UK Brain Bank criteria, and informed consent. PD cases were enrolled sequentially as they were seen at the UAB clinic. Controls consisted of patients’ spouses and community volunteers. Inclusion criteria for controls were informed consent and self-report of not having PD, stroke, ataxia, multiple sclerosis, Alzheimer’s disease, dementia, dystonia, autism, bipolar disorder, amyotrophic lateral sclerosis, or epilepsy (hence, neurologically healthy controls, NHC).

Enrollment occurred in two waves: July 2015–July 2017 (*N* = 316 PD and 181 NHC) and October 2018–March 2020 (*N* = 665 PD and 304 NHC). The sample size was determined by the arrival of the COVID-19 pandemic in the Deep South when we stopped enrolling.

Subjects were asked to fill out two questionnaires: the Environmental and Family History Questionnaire (EFQ) and the Gut Microbiome Questionnaire (GMQ, the source for gastrointestinal data), self-administered with no interference from the research staff. In the first enrollment wave, subjects completed the questionnaires in the clinic. In the second wave, subjects took the questionnaires home, and 74% of PwP (*N* = 492) and 77% of NHC (*N* = 234) completed and returned them. The final analytic sample size with completed questionnaires was 808 PD and 415 NHC.

Questions that were asked to collect the data used in this analysis are shown in Supplementary Material. Quality control checkpoints were implemented at every step possible during data collection, data entry, and data analysis. Positive family history was defined as having at least one first- or second-degree relative with PD; negative family history was the absence of PD in first- and second-degree relatives; and an indication of family history without specifying the degree was classified as unknown. MTBI/concussion events (*N* = 19) and exposures to pesticides/herbicides (*N* = 5) that occurred in the same year or after the onset of PD were not counted as events. When limiting MTBI/concussions to incidences that occurred more than 10 years prior to PD onset, the incidences within 10 years were treated as no event.

The number of subjects with data for each item can be extracted from the full dataset provided in Supplementary Material and are also given for each test in the Tables. Each data point was taken from a distinct sample (subject). For software, the URLs and versions used are given in Supplementary Material. The code for data analysis is available on [https://zenodo.org/record/10072984].

Differences in frequencies of features (constipation, RBD, weight loss) in PD vs. NHC were tested using Yates continuity-corrected OR, using the function ‘Prop.or‘ from the pairwiseCI R package specifying “CImethod = ‘Woolf”.

Association between risk factors and disease was tested under the null hypothesis of no difference and the alternative hypothesis that risk factor is associated with increased risk. There was no reason to think or test if they may be associated with a reduced risk of PD because these risk factors were chosen specifically because they have been robustly associated with increased PD risk in other populations. Given that the alternative hypothesis was one-sided (positive direction), tests were conducted one-sidedly. To test each risk factor individually, OR, lower bound (LB) of 95% confidence intervals (CI), and one-sided *P* values were calculated with no covariate adjustment from logistic regression models using ‘glm‘ in R. To assess the strength and significance of each risk factor in the presence of, and adjusted for, other risk factors we used multivariable logistic regression analysis, where we included all relevant risk factors in the regression model, as shown in Eqs. (1) and (2):

1 $$\begin{array}{ll}{\rm{PD}}/{\rm{Control}}|{\rm{Male}} \sim \left[{\rm{age}},{\rm{family}}\; {\rm{history}},{\rm{MTBI}}/{\rm{concussion}},\right.\\{\rm{repeated}}\; {\rm{blows}}\; {\rm{to}}\; {\rm{head}}\; {\rm{in}}\; {\rm{sports}}\; {\rm{or}}\; {\rm{military}},\\ \left.{\rm{pesticide}}/{\rm{herbicide}}\; {\rm{exposure}},{\rm{military}}{\hbox{-}}{\rm{related}}\; {\rm{chemical}}\; {\rm{exposure}}\right]\end{array}$$

2 $${\rm{PD}}/{\rm{Control}}|{\rm{Female}} \sim [{\rm{age}},{\rm{family}}\; {\rm{history}},{\rm{MTBI}}/{\rm{concussion}},{\rm{pesticide}}/{\rm{herbicide}}\; {\rm{exposure}}]$$

Since the male sex was collinear with repeated blows to the head, and military-related chemical exposure, the sexes were analyzed separately. One-sided *P* value was calculated using ‘glm‘ specifying ‘family = “binomial”‘ in R. Adjusted odds ratio was calculated using equation ‘exp(*x*)‘ in R where *x* is a vector of coefficients extracted from ‘glm‘ in R. The lower bound for the one-sided 95% CI was calculated by taking the lower bound of a two-sided 90% Wald CI, using ‘exp(confint.default(*x*, level = 0.9))‘ in R where x is a ‘glm‘ object from running the multivariable models, and Level=0·9 specifies the two-sided 90% interval.

Population attributable fraction was calculated using the Miettinen formula^3^:

3 $${\rm{PAF}}={\rm{p}}{\rm{c}}(1-1/{\rm{RR}})$$

Where p_c_ is the prevalence of risk factors in cases and RR is the relative risk approximated by OR under the rare disease assumption. To adjust PAF for potential confounding, we used adjusted OR values derived from multivariable analysis. For the 95% CI, we used the function ‘AFglm‘ from the AF R package on the regression models, specifying ‘case.control = TRUE‘, using a sandwich estimator for the variance of PAF.

To assess the fraction of disease attributed to multiple risk factors, we calculated joint PAF (also known as summary PAF). Prior to calculating joint PAF, in order to meet the assumption of absence of interaction, we tested for pair-wise interaction between the three risk factors associated with PD in males. We used Eq. (1) and added an interaction term. There was no evidence of interaction in this dataset. The interaction *P* values were *P* = 0.99 for [pesticide/herbicide exposure*military-related chemical exposures], *P* = 0.99 for [military-related chemical exposures*repeated blows], and *P* = 0.56 for [pesticide/herbicide exposure*repeated blows to head]. Having established no interaction, we used Eq. (4) to calculate joint PAF.

4 $${\rm{PAF}}{\rm{Joint}}{\rm{|Males}}=1-\Pi {\rm{i}}(1-{\rm{PAFi}})$$

$${\rm{PAFJoint|Males}}=1-[(1-{{\rm{PAF}}}_{{\rm{military}}{\hbox{-}}{\rm{related}}\; {\rm{chemicals}}})* (1-{{\rm{PAF}}}_{{\rm{repeated}}\; {\rm{blows}}\; {\rm{to}}\; {\rm{head}}})* (1-{{\rm{PAF}}}_{{\rm{pesticide}}/{\rm{herbicide}}})]$$

The upper and lower bounds of 95% CI of joint PAF were calculated using Eq. (4) with the set of three upper bounds and three lower bounds of the individual adjusted PAFs.

To assess potential confounding by race/ethnicity, and spousal PD-NHC pairs vs. non-spousal singleton PD and NHC, we first excluded Hispanic and non-White individuals, then separated them into two non-overlapping groups of spousal pairs (106 PD, 106 NHC) and singletons (657 PD, 291 NHC), and repeated the analyses as described above, except Firth’s penalized logistic regression (logistf R) was used for military-related chemical exposures in singletons because the count for exposed NHC males was zero (Supplementary Material). Subsequently, the multivariable analysis for singleton men had to be analyzed using the Firth penalty because one variable had a zero count. And because we used adjusted OR from multivariable analysis for PAF, the effect sizes used in PAF in singleton men are adjusted for that one zero count.

### Supplementary information

Supplementary Material

Related Manuscript File

## Data Availability

Data are publicly available without restriction. The entire dataset at the individual level, de-identified, is provided in Supplementary Material. As per Human Subject Research Privacy considerations, any subject over age 90 years was denoted as 90 in publicly shared data although true age was used in this analysis. For software used, the URLs and versions are given in Supplementary Material.

## Code availability

The code for reproducing the results can be found on [https://zenodo.org/record/10072984].
