# Supplementary material for: Population fraction of Parkinson’s disease attributable to preventable risk factors
Source: NPJ Parkinsons Dis. 2023 Dec 5;9:159. doi: 10.1038/s41531-023-00603-z (PMC10698155; doi:10.1038/s41531-023-00603-z)
Supplement: Supplementary file 1 — Supplementary Material [file 41531_2023_603_MOESM1_ESM.pdf]

Population fraction of Parkinson's disease attributable to preventable risk factors

Haydeh Payami*, Gwendolyn Cohen, Charles F Murchison, Timothy R Sampson, David G Standaert, Zachary D Wallen

*Corresponding author

Supplementary Material

Data	page 2-39
Questions	page 40-41
Race and spousal relationship	page 42-45
Software	page 46

Supplementary Material/Data. Individual level data on 1223 subjects used in study. Consent has been obtained for sharing of individual-level data.

Case status: PD: Parkinson's disease. NHC: neurologically healthy control

Race: A: Asian. AA: American Indian/ Alaskan Native. B: Black or African American. M: more than one race. W: white

RBD: Rem sleep behavior disorder

Family history PD degree: First: at least one first degree relative has PD. Second: no first degree, at least a second degree relative has PD. Third+: no first or second, but a relative at third or higher degree has PD

Subject ID	Case status	spouse	Sex	Age	PD onset age	RBD	MTBI/concussion	MTBI/concussion age	Repeated blows to head	Chemical warfare exposure	Pesticide/ herbicide exposure	Pesticide/ herbicide Exposure duration, yrs	State subject was born	State current residence	Hispanic or Latino	Race	Jewish ancestry	Family history PD	Family history PD degree	Lost 10 lbs last year	Constipation
DP014	PD	DC004	M	77	66	N	N		N	N			AL	AL	N	W	N	Y	first	N	N
SC0052	NHC		F	65			N							AL	N	W		Y	third+	N	Y
DP576	PD		M	67	65	N	N		N	N	N		AL	AL	N	W	N	N		N	N
DC313	NHC		M	65		N	N		N	N	N		AL	AL	N	W	N	N		N	N
DP528	PD		M	68	67	N	Y	18	N	N	N		OK	AL		AA	N	N		Y	Y
DP137	PD	DC056	M	64	57	N	N		Y	N	N		IL	AL	N	W	N	N		Y	Y
DP454	PD		M	67	58	N	Y		N	N	N		AL	AL	N	W	N	N		Y	Y
DP042	PD	DC020	M	74	55	N	Y after onset	70	N	Y	N		AL	AL	N	W	N	Y		N	Y
DP043	PD		F	63	62	N	N			N	N		GA	AL	N	W	N	Y	first	Y	N
DP088	PD	DC038	F	82	80	N	N		N	N	N		AL	AL	N	W	N	Y	first	N	N
DP095	PD	DC041	F	66	54	N	N		N	N	Y	11	NC	AL	N	W	N	N		Y	Y
DP437	PD	DC138	M	72	68	N	N		Y	N	N		AL	AL	N	W	N	N		Y	N
SP0190	PD		M	50	35		N							AL		W		N		N	N
DP027	PD		M	66	65	Y	N		N	N	N		IL	AL	N	W	N	Y	second	N	Y
SC0556	NHC		M	65			Y	8	Y	N	N			AL	N	W	N	N		N	N
DP052	PD		F	42	40	N	N		N	N	N			AL	N	B	N	N		N	Y
SP0002	PD		M	43	23		Y	17						AL	N	W		Y	third+	N	N
DC111	NHC	DP342	M	70		N	N		N	N	N		AL	AL	N	W	N	N		Y	N
SC0025	NHC		F	61			N							AL	N	W		N		N	N
DP078	PD		M	68	63	N	N		N	N	N		AL	AL	N	W	Y	N			

SC0101	NHC		F	59			N							AL	N	W		N		N	N
DP456	PD		F	68	60	N	Y after onset	66	N	N	Y	26	MS	MS	N	W	N	N		Y	
DP237	PD		M	79	74	Y	N		N	N	N			FL	N	W	N	N			N
DP225	PD	DC075	M	86	81	N				N	Y	17	MS	AL	N	W	N	N		N	Y
DP032	PD	DC019	M	65	60	N	Y	9	N	N	N		CA	AL	N	W	N	N		N	N
DP577	PD		F	62	35	N	N		N	N	N		AK	AL	N	AA				N	
DP170	PD		M	47	45	N	N		N	N	N		AL	AL	N	W	N	N		Y	Y
DC125	NHC	DP373	F	66		N	N		N		N		FL	AL	N	W	N	N		Y	N
DP393	PD		M	66	56	Y				N	N		KY	AL	N	W	N	N		N	Y
SP0960	PD		M	61	59		N		N					MS	N	W	N	N		N	Y
SC0633	NHC		M	60			N		N	N	N			MS	N	W	N	N		Y	N
DP616	PD		F	69	68	Y	N		N	N	Y	40		AL	N	W	N	N		N	N
DP082	PD		M	82	71	N	Y after onset	81	N	N	Y	2	IN	AL	N	W	N	Y	first	N	N
DP185	PD		F	56	48	N	N			N	N		TN	AL	N	W	N	N		Y	N
SP1100	PD		M	75	69		N			N	N			AL	N	W	N	N		N	N
DP181	PD		M	74	65	N	N		N	N	N		MS	MS	N	W	N	Y	third+	N	Y
DP063	PD		M	70	60	N	N		N	N	N		IL	AL	N	W	N	N		N	N
DC137	NHC	DP436	M	68		N	N		N	N	Y	62	AL	AL	N	W	N	N		N	N
DC221	NHC		M	65		N	Y	54	N	N	N		Not US	AL	N	W	N	N		N	N
DP280	PD		M	62	55	N	Y	14	Y	N	N		AL	AL	N	W	N	N		N	N
DP094	PD		M	70	49	N	N			N	N			AL	N	W	N	N		N	Y
DC203	NHC		F	64		N	N		N	N	N		AL	AL	N	W	N	N		N	N
SP0035F51	PD		F	64	48		Y	45						AL	N	W		N		N	Y
DP263	PD		M	75	70	N	Y	18	Y	N	Y	8	AL	AL	N	W	N			N	Y
DP222	PD		M	71	59	N	N		N	N	N		Not US	AL	N	W	N	N		N	N
DP646	PD	DC254	M	74	70	N	N		N	N	N		TX	AL	N	W	N	N		Y	N
DP149	PD		F	75	59	N	Y	6	N	N	N		AL	MS	N	W	N	Y	first	Y	N
SP1001	PD		M	63	48		N		N	N	N			AL	N	W	N	N		N	Y
DP169	PD	DC060	M	71	68	N	N		N	N	N		AL	AL	N	W	N	N		N	N
SC0562	NHC		M	63			N		N	N	N			AL	N	W	N	N		N	N
SP0261	PD		F	65	52		Y	12	N					Al	N	W	N	N		N	Y
DP607	PD		F	69	63	N	N		N	N	N		CT	TN	N	W	N	N		N	Y

SC0537	NHC		F	53			N			N	N			NC	N	W	N	Y	second	N	N
SP1016	PD		M	63	58		N		N	N	N			AL	N	W	N	N		Y	N
SC0620	NHC		M	69			N		N	N	N			AL	N	W	N	N		N	N
SP0572	PD		F	74	62		N		N	N	N			AL	N	W	N	N		N	Y
DC224	NHC		M	78		N	N		N	N	N		AL	AL	N	W	N	N			N
SC0266	NHC		F	78			N		N	N	Y			AL	N	W	N	N		N	Y
DC217	NHC		F	61		N	N		N	N	N		AL	AL	N	W	N	N		N	N
DP115	PD		M	62	59	N	N		N	N	N		Not US	AL	N	A	N	N		N	Y
SP1075	PD		F	68	55		N		N	N	N			AL	N	W	N	N		N	N
SP0009	PD		F	60	45		N							AL	N	W		Y	second	N	N
DC118	NHC	DP392	F	68		N	N		N	N	N		AL	AL	N	W	N	Y	first	N	N
SP1043	PD		M	66	62		Y	21	Y	Y	Y			AL	N	W	N	Y	first	Y	N
DP256	PD		M	79	71	N	N		N	N	N		AL	AL	N	W	N	Y	first	N	N
SP0041F37	PD		F	78	71		Y							AL	N	W		Y	third+	N	
DP394	PD		M	90	50	N	N		N	Y	N		Not US	AL	Y	W	N			N	Y
SP0952	PD		M	42	42		N		N	N	N			AL	N	W	N	N		Y	N
SP0237	PD		M	67	63		N							AL	N	W		N		Y	N
SP0233	PD		M	64	59		N							AL	N	W		N		N	N
SP0168	PD		F	69	61		N							AL	N	W		N		N	N
DP122	PD	DC048	M	63	51	N	N		N	N	N		AL	AL	N	W	N	Y	first	Y	N
SP0222	PD		M	73	39		N							GA	N	W		Y	second	N	Y
SP0994	PD		F	56	35		N		N	N	Y			TN	N	W	N	N		N	N
DP492	PD	DC154	M	67	59	N	Y	10	N	N	N		AL	AL	N	W	N	N		N	Y
DP627	PD		M	69	54	N	N			N	N		AL	AL	N	W	N	N		N	Y
DP031	PD	DC018	M	81	66	N	N		N	N	Y	18	AL	AL	N	W	N	Y	first	N	Y
DP022	PD	DC013	M	63	50	N	N		Y	N	Y		PA	AL	N	W	N	N		Y	N
SC0009	NHC		F	71			N							AL	N	W		N		Y	N
SC0098	NHC		F	65			N							AL	N	W		N			
DP066	PD		M	79	78	N	N		N	N	N		AL	AL	N	W	N	Y	first	N	N
DP274	PD		M	71	70	N			N	N	N		WA	MS	N	W	N	N		N	Y
SC0074	NHC		M	80			N							AL	N	W		N		N	Y
SC0028	NHC		F	69			N							MS	N	W	Y	N		N	Y

DP157	PD		F	71	62	N	N		N	N	Y	3		AL	N	W				N	N
DC140	NHC	DP448	F	56		N	N			N	N		GA	AL	N	W	N	N		N	N
SC0613	NHC		F	62			Y	10	N	N	Y				N	W	N	N		N	N
DP560	PD		M	72	60	Y	Y		Y	Y	N			AL	N	W	N	Y	first	N	N
DP428	PD		F	60	56	N	N		N	N	Y	9	GA	GA	N	W	N	Y	first	N	N
DP295	PD	DC099	M	59	52	N	N		Y	N			DE	AL	N	W	N	Y	second	N	N
DC113	NHC	DP346	F	71		N	N			N			AL	AL	N	W	N	N		Y	N
SP1040	PD		F	73	71		Y	29	N	N	Y			AL	N	W	N	N		N	Y
SP0118	PD		M	67	59		N							MS	N	W		Y	second	N	N
SP0077	PD		M	69	64		Y	60						AL	N	W		N		N	N
DP338	PD		M	57	40	N	N			N				AL	N	W				N	
DP533	PD		F	65	63	N	N		N	N	N		AL	AL	N	B	N	N		N	Y
SC0536	NHC		M	53			N		N	N	N			NC	N	W	N	Y	second	N	N
DP146	PD		F	76	63	N	N		N		Y	16	TX	AL	N	W	N	Y	first	N	N
SC0541	NHC		F	54			N		N	N	N				N	W	N	N		N	N
SP1023	PD		M	71	67		Y after onset	68	N	Y	N			AL	N	W	N	Y	second	N	Y
SP0198	PD		M	79	72		N							AL	N	W		N		N	N
SP0991	PD		M	77	71		N		N	N	N			AL	N	W	N	N		Y	N
SP0986	PD		F	62	55		N		N	N	N			AL	N	W	N	N		Y	Y
DC098	NHC	DP289	F	76		N	N		N	N	N		UT	AL	N	W	N	N		N	
DP029	PD		M	65	59	N	N		N	N	N		TX	AL	N	W	N	N		N	Y
DP502	PD		F	69	61	N	N		N	N	Y	20	AL	AL	N	W	N	Y	second	N	Y
DP466	PD		M	78	75	N	N		N	N	N		MN	AL	N	W	N	Y	first	N	Y
SP1065	PD		M	72	61		N		N	N	Y			AL	N	W	N	N		N	Y
SP0272	PD		F	64	63		N		N	N	N			AL		W	N	N		N	N
DP598	PD		F	47	44	N	N		N	N	N		AL	AL	N	W	N	N		N	N
SC0095	NHC		F	64			N							AL	N	W		N		N	Y
DC268	NHC		F	56		N	N		N	N	N		TN	AL	N	W	N	N		Y	N
DP564	PD	DC263	M	63	56	N	N		N	N	N		AL	AL	N	W	N	Y	first	N	Y
DP401	PD	DC119	M	72	69	N	Y	63	N	N	Y			AL	N	W	N	N		N	N
DP455	PD		M	78	75	N	Y	70	N	N	N		IL	AL	N	W	N	N		N	N
SP0262	PD		M	75	72		Y	40	N	N	N			AL	N	W		N		N	Y
DC249	NHC	DP547	F	46		N	N		N	N	N		TX	AL	N	W	N	N		N	N

DP342	PD	DC111	F	59	50	N	N		N	N	N		PA	AL	N	W	N	N		N	N
SP1074	PD		F	68	65		N		N	N	N			AL	N	W	N	Y	first	N	N
DP253	PD	DC083	M	68	66	N	N		N	N	N		AL	AL	N	W	N	N		N	N
SP0180	PD		M	68	58		N							AL	Y	W		N		N	N
DC005	NHC	DP007	F	56			N			N	N			AL	N	W					
SC0609	NHC		F	73			N		N	N	N			AL	N	W	N	N		N	N
SP0128	PD		F	67	59		N							FL	N	W	Y	Y	second	Y	Y
SP1013	PD		M	75	60		N		Y	N	Y			AL	N	W	N	N		N	N
SC0018F50	NHC		F	65			N							AL	N	W		N		N	N
SC0147	NHC		F	57			N		N	N	N			AL	N	W	N	N		N	N
SP0706	PD		M	65	63		Y	16	Y	Y	Y			AL	N	W	N	N		N	N
DC094	NHC	DP279	F	72		N	N			N	N		AL	MS	N	W	N	N		N	N
DP440	PD		F	74	66.5	N	N		N	N	N		LA	AL	N	W	N	N		N	N
DC177	NHC		F	49		N	N		N	N	Y	5	TN	AL	N	W	N	N		N	N
SP1039	PD		M	83	66		N		Y	N	N			AL	N	W	N	N		N	Y
DC075	NHC	DP225	F	82		N	N		N	N	N		MS	AL	N	W	N	N		N	Y
SP1107	PD		M	64	50		N		N	N	N			AL	N	W	N	Y	second	Y	N
DP548	PD		M	66	64	N	N		N	N	Y	28	AL	AL	N	W	N	Y	second	Y	Y
DP003	PD		F	57	55	N	N		N	N	N		AL	AL	N	W	N	Y	first	Y	Y
SP0186	PD		M	57	48		Y	14						AL	N	W		Y	second	Y	N
DP303	PD		F	59	48	N	N			N	Y		AL	AL	N	W	N	N		N	N
DP071	PD	DC034	F	79	79		N		N	N	N		AL	AL	N	W	N	Y	first	N	
SP0001	PD		M	55	40		Y	16						AL	N	W		Y	second	N	Y
DP143	PD		M	59	50	N	Y after onset	53	N	N	N		Not US	AL	Y		N	N		N	N
DP582	PD		M	77	72	N	N		N	N	Y	76	AL	AL	Y	W	N	N		Y	N
SP0189	PD		F	66	62		N							AL	N	B		N		N	N
DC067	NHC	DP198	M	70		N	Y	15		N	Y	21	GA	AL	N	W	N	N		Y	N
SP0888	PD		M	74	52		N		N	Y	N			AL	N	W	N	N		N	N
DC054	NHC	DP135	F	75		N	N		N	N	N		GA	GA	N	W	N	N		N	N
SP0220	PD		M	86	80		N							AL	N	W		Y	first	N	Y
SC0055	NHC		F	61			N							AL	N	W		N		N	N
DC104	NHC		F	65		N	N		N	N	N		AL	AL	N	W	N	N		N	N
SP0961	PD		M	57	51		N		Y	N	N			AL	N	W	N	N		Y	N

DC023	NHC	DP049	F	73		N	Y	13	N	N	Y	11	TX	MS	N	W	N	N		Y	N
DP100	PD	DC044	M	69	67	N	N		N	N	N		NY	AL	N	W	N	N		Y	Y
SP1068	PD		F	73	68		N		N	N	N			MS	N	W	N	Y	first	Y	N
SC0022F51	NHC		F	64			N							FL	N	W		N		N	N
DP068	PD		M	72	65	N	N		N		N		NY	AL	N	W	N	Y	third+	N	N
DP188	PD	DC064	M	64	53	N	N		N	N	N		AL	AL	N	W	N	Y	first	N	Y
SC0110	NHC		F	77			N							AL	N	W		N		N	N
SP0156	PD		M	75	64		N							AL	N	W		N		N	Y
SC0595	NHC		F	71			Y	4	N	N	N			AL	N	W	N	N		N	N
DC048	NHC	DP122	F	57		N	N			N			AL	AL	N	W	N	N		N	N
DP060	PD	DC029	M	45	39	Y	Y	5	N	N	N		AK	AL	Y	W	N	N		N	Y
DC151	NHC		M	47		N	N		N	N	N		AL	AL	N	W	N	Y	second	N	N
SP1101	PD		M	74	65		N		N	N	N				N	W	N	Y	first	N	N
DP261	PD		M	56	44	N	N		N	N	N		AL	AL	N	W	N	Y	first	N	N
SP0111	PD		F	82	74		N							AL	N	W				N	Y
DC149	NHC		F	49		N	N		N	N	N		VA	AL	N	W	N	Y	second		N
DP299	PD		M	53	49	N	N		N	N	Y	35	AL	AL	N	W	N	N		Y	Y
SP0310	PD		F	68	42		N		N	N	N				Y	W	N	Y	third+	N	Y
DP177	PD		M	75	73	N	N			N			AL	AL	N	W	N	Y	first	N	Y
DP096	PD	DC042	M	66	62	N	Y	17	Y	N	N		ME	AL	N	W	N	N		N	Y
SC0033	NHC		M	75			N							AL	N	W		N		N	N
DP162	PD		M	71	69	N	N		Y	N	Y	8	MS	MS	N	M	N	N		N	N
DC077	NHC		F	68		N	N		N	N	N		AL	AL	N	W	N	N		N	
SP0043F37	PD		F	78	64		N							AL	N	W		N		N	N
DC231	NHC		F	73		N	N		N	N	N		AL	AL	N	W	N	N		Y	N
DP407	PD		F	70	53	N	Y after onset	66	N	N	Y	18		AL	N	W				N	N
SP0947	PD		M	70	56		N		N	N	N			AL	Y	W	N	Y	first	N	N
DP189	PD	DC065	M	75	62	N	N		N		N		AL	AL	N	W	N	N		N	Y
SP0177	PD		M	69	62		N							MS	N	W		N		N	Y
SP0208	PD		M	81	80		N							AL	N	W		N		N	Y
SP0628	PD		M	72	69		N		N	N	N			AL	N	W	N	N		N	N
SC0076	NHC		F	69			N							MS	N	W		N		N	N
DC120	NHC	DP402	M	69		N	N		N	N	N		AL	AL	N	W	N	N		N	N

SC0021M3 6	NHC		M	80			N							AL	N	W		N		N	Y
DP596	PD		M	84	76	N	N		Y	N	Y	60	IN	AL	N	W	N	N		N	N
DP312	PD		M	69	64	N	N		N	Y	N		AL	AL	N	W	N	N		N	Y
DP265	PD		M	79	62	N	N		N	N	N		AL	AL	N	W	N	N		N	N
SP0270	PD		F	48	41		N		N	N	N			AL	N	W	N	N		Y	N
SP0943	PD		F	71	65		N		N	N	N			FL	N	W	N	N		N	N
SP0945	PD		F	57	46				N	N	Y			AL	N	W	N	N			
SC0007	NHC		F	61			N							AL	N	W		N		N	N
SP0253	PD		F	65	52		N		N	N	Y			Ms	N	W	N	N		N	N
SP1053	PD		M	76	74		N		N	N	N			AL	N	W	N	N		N	Y
SP0049M35	PD		M	80	69		N							AL	N	W		N		N	
DC070	NHC	DP208	F	68		N	N		N	N	N		KY	AL	N	W	N	Y	second	N	N
DP339	PD		M	70	65	N	N		N	N	N		AL	AL	N	W	N	N		Y	Y
SP0062	PD		F	66	57		N							AL	N	W		N		N	N
DP535	PD	DC223	M	77	72	N	N		N	N	N		DE	AL	N	W	N	N		Y	N
DP101	PD		M	54	38	Y	N		N	N	N		AL	AL	N	B	N	N		Y	Y
DP211	PD		M	58	40	N	Y	35	Y	N	Y			AL	Y	W	N	N		Y	Y
DP305	PD		F	68	64	N	Y	59	N	N	N		AL	AL	N	W	N	N		N	N
DC038	NHC	DP088	M	83		N	N		N	N	N			AL	N	W	N	Y	first	N	N
SP0112	PD		F	58	55		N							AL	N	W		N		N	Y
SC0050	NHC		F	53			N							AL	N	W		N		N	N
SC0545	NHC		M	71			N		N	N	N			AL	N	W	N			N	Y
DC081	NHC	DP333	F	78		N	N		N	N	N		AL	AL	N	W	N			N	N
DP050	PD	DC024	M	63	60	Y	N		N		Y		AL	AL	N	W	N	Y	first	Y	Y
SC0031	NHC		F	69			N							AL	N	W		N		N	Y
DC312	NHC	DP639	F	69		N	N		N	N	N		TN	AL	N	W	N	N		N	N
SP1087	PD		M	70	66		N		N	N	N			AL	N	W	N	N		Y	N
SC0008	NHC		M	67			N							AL	N	W		N		N	Y
DP569	PD		F	66	57	N	N		N	N				AL		B		N		N	Y
DP651	PD		M	77	74		N		N	N	N			AL	N	W	N	N		Y	Y
SC0589	NHC		F	68			N		N	N	N			AL	N	W	N	N		N	Y
SC0128	NHC		F	71			N							AL	N	W		N		N	Y
DP379	PD		M	63	59	N	N		N	N	Y	10	TN	MS	N	W	N	N		N	N

DP254	PD		M	72	70	N	Y	10	N	N	Y	2	TN	AL	N	W	N	N		Y	N
DP075	PD		M	50	42	N	N		N		Y	12	MS	MS	N	W	N	N		N	Y
DP615	PD	DC287	F	59	50	N	N		N	N	N		AL	FL	N	W	N	N		N	Y
DC290	NHC	DP620	F	64		N	N		N	N	N		AL	AL	N	W	N	N		N	Y
SC0544	NHC		F	62			N		N	N	N			AL	N	W	N	Y	second	N	N
SP0517	PD		M	76	75		N		N	N	N			AL	N	W	N	Y	third+	N	Y
DC241	NHC		F	67		N	N		N	N	N			AL						N	N
DP416	PD		M	71	65	N	N		N	N	N		GA	AL	N	W	N	Y	first	Y	N
DC173	NHC	DP516	F	66		N	N		N	N	N		AL	AL	N	W	N	N		Y	N
SP0067	PD		M	63	47		Y after onset	56						AL	N	W		N		N	Y
DP012	PD		M	55	40	N	N		N	N	N		AL	AL	N	B	N	N		Y	Y
DC211	NHC		F	45		N	N		N	N	N			AL	N	W	N	N		N	N
DP361	PD		F	55	38	N	N		N	N	Y	14	AL	MS	N	W	N	Y	first	N	Y
DP634	PD		F	59	54	N	N		N	N	N		AL	AL	N	W	N	N		Y	N
SP1038	PD		M	81	60		N		N	N	Y			AL	N	W	N	N		N	N
SC0120	NHC		F	54			N							AL	N	W		N		Y	N
SP0068	PD		F	77	74		Y	74						AL	N	W		N		Y	Y
SP0193	PD		F	65	48		N							AL	N	W		Y	third+	Y	Y
SP0259	PD		F	76	59		N		N	N	N			Al	N	W	N	N		N	N
DC026	NHC	DP053	F	72		N	N		N	N	N		TN	AL						N	Y
DC083	NHC	DP253	F	68		N	N		N	N	N		AL	AL	N	W	N	N		N	N
DP197	PD		M	77	70	N	N			N	Y		AL	AL	N	W	N	N		N	Y
SP0206	PD		F	72	69		N							AL	N	W		N		N	N
DP206	PD		M	63	26	N	Y		Y	N	Y	25	AL	AL	N	W	N	Y	third+	N	N
DP024	PD		F	78	74	N	N		N	N	N		AL	AL	N	W	Y	N		N	N
SP0194	PD		F	64	63		Y	15						AL	N	W		N		N	N
DC223	NHC	DP535	F	77		N	N		N	N	N		PA	AL	N	W	N	N		N	N
DC049	NHC		M	64		N	N			N	N		MS	TX	N	W	N	N		N	N
SP0150	PD		F	75	70		N							AL	N	W		N		Y	N
DP527	PD	DC198	M	56	55	N	Y	21		N	Y	11	AL	AL	N	W	N	N		Y	N
SC0005	NHC		F	74			N							AL	N	W		N		N	N
DP558	PD		F	77	70	N	N		N	N	N		TN	AL	N	W	N	N		N	N
SP0938	PD		M	65	64		Y	16	N	N	Y			MS	N	W	N	Y	second	Y	N

DP147	PD		M	70	54	N	N		N	N	N		MS	MS	N	W	N	N		N	N
DP154	PD		M	67	66	Y	N		N	N	Y	12	AL	AL	N	W	N	N		N	N
DP139	PD	DC058	M	69	62	N	N		N	N	N		AL	AL	N	W	N	N		N	N
DP391	PD		M	82	69	N	Y	25	N	N	Y	4	CA	AL	N	W	N	N		Y	N
DP413	PD		M	71	61	N	N		N	N	Y	70	MS	MS		W	N	N		N	N
DP175	PD		F	66	48	N	Y		N	N	N		AL	AL	N	W	N	Y		Y	N
SC0023F37	NHC		F	78			N							AL	N	W		N		N	N
DC274	NHC		F	50		N	N		N	N	Y	13	IN	AL	N	W	N	N		N	N
SC0069	NHC		F	67			N							AL	N	W		N		N	N
DP336	PD		M	55	32	N	N		N	N	N		AL	AL	N	W	N	Y	first	N	N
DP121	PD		M	69	61	N	N		Y	N	N		AL	AL	N	W	N	N		N	N
SC0538	NHC		M	54			N		N	N	N			AL	N	W	N	Y	second	N	N
SC0118	NHC		F	62			N							GA	N	W		N		N	
SP0169	PD		F	72	66		N							MS	N	W		Y	second	N	Y
SP0250	PD		F	70	67		N							AL	N	W		N		Y	Y
SP0051	PD		M	66	57		Y after onset	63						AL	N	W		N		Y	Y
SC0014F45	NHC		F	70			N							AL	N	W		Y	first	N	N
DP445	PD		F	66	50	N	N		N	N	N		GA	GA	N	W	N			N	N
DP316	PD		F	54	49	N	N		N	N	N		TN	AL	N	W	N	Y	second	Y	N
DC163	NHC		M	80		N	Y	56	N	N	N		AL	AL	N	W	N	N		N	N
DP009	PD	DC006	F	37	35	N	N		N	N	N		AL	AL	N	W	N	Y	second	N	N
DP200	PD		M	63	60	N	Y	15	Y	N	N		MS	MS	N	W	Y	N		Y	N
SP0751	PD		M	64	64		N		N	N	N			MS	N	W	N	N		Y	N
SC0034	NHC		F	73			N							AL	N	W		N		N	N
DP058	PD		F	52	51	N	N		N	N	N		AL	AL	N	W	N	N		Y	Y
SC0035	NHC		F	61			N							AL	N	W		N		N	N
SP0124	PD		F	62	54		N							AL	N	W		N		N	Y
SP1069	PD		F	73	65		N		N		Y			AL	N	W	N	N		Y	N
SP0981	PD		M	75	63		Y after onset	73	N	N	Y			AL	N	W	N	N		N	N
DC271	NHC		F	65		N	N		N	N	N		AL	AL	N	W	N	Y	first	Y	N
DC042	NHC	DP096	F	62		N	Y	25	N	N	N		AL	AL	N	W	N	N		N	N
SP0147	PD		F	64	53		Y	16						AL	N	W		N		N	Y
SP0200	PD		M	86	84		N							AL	N	W		N		N	N

DC291	NHC		F	67		N	N		N	N	N		TN	AL	N	W	N	N		N	N
DC016	NHC	DP026	M	70										AL							
DP219	PD		F	78	48	N	N		N	N	Y	12	AL	AL	N	W	N	Y	first	N	Y
SC0463	NHC		M	64			N		N	N	N			AL	N	W	N	N		N	N
SC0580	NHC		M	77			Y	6.5	N	N	Y			AL	N	W	N	N		N	N
DP516	PD	DC173	M	68	63	N	N		N	N	N		AL	AL	N	W	N	N		N	N
DP231	PD		M	77	68	N								AL	N	W					Y
SP0176	PD		F	90	83		N							AL	N	W		Y		Y	N
SC0629	NHC		F	70			N		N	N	Y			MS	N	W	N	N		Y	Y
DC167	NHC		F	69		N				N	N			AL	N	W	N	N		Y	N
SP0203	PD		M	76	66		N							AL	N	W		N		N	N
DP483	PD		M	70	65	Y	Y	50	N	N	N			AL	N	W	N	N		N	N
DP045	PD	DC021	M	68	56	N	N		N	N	Y	68	MS	MS	N	W	N	N		N	Y
DP193	PD		M	67	54	N	N			N	N		AL	AL	N	W	N	N		N	Y
DC216	NHC		F	69		N	N		N	N	N		GA	AL	N	W	N	N		Y	N
DP545	PD		M	78	75	N	N		N	N	Y	35	GA	AL	N	W	N	Y	first	N	N
SP0990	PD		F	56	45		N		N	N	N			AL	N	W	N	N		N	Y
SP1089	PD		M	69	60		N		N	N	N			AL	N	W	N	Y	first	N	N
DC128	NHC	DP107	F	73		N	N		N	N	Y	3	AL	AL	N	W	N	N		N	Y
DP362	PD		F	67	56	N	N		N	N	N		MS	MS	N	W	N	N		N	Y
DP313	PD		F	77	72	N	N		N	N	N		MI	AL	N	W	N	Y	second	N	N
DP449	PD	DC141	F	74	67	N	N		N				AL	AL	N	W	N	N		N	Y
DP625	PD	DC292	M	87	83	N	N		N		N			AL		W	N	Y	first	Y	Y
SC0036	NHC		F	66			N							AL	N	W		N		N	N
DC085	NHC	DP252	F	63		N	N		N	N	N		TN	AL	N	W	N	Y	first	N	Y
SC0044	NHC		F	77			N							AL	N	W		N		N	Y
SP1098	PD		F	57	49		N		N	N	N			MS	N	W	N	N		N	Y
DP176	PD		M	71	60	N	N		N	N			TN	AL	N	W	N	Y	first	N	Y
SP0266	PD		F	62	50		N		N	N	Y			Al	N	W	Y	Y	first	N	Y
SC0089	NHC		M	64			N							AL	N	W		N			N
DC126	NHC	DP405	F	70		N	N		N	N	N		UT	AL	N	W	N	Y	second	N	Y
DC267	NHC	DP574	M	66		N	N		N	N	N		AL	AL	N	W	N	N		N	N
DP488	PD		F	48	40	N	Y	8	N	N	Y	18	MS	MS		W	N	Y	second	N	Y

SP0013	PD		M	64	62		N							AL		W		N		N	N
DP602	PD		M	81	62	Y								AL	N	W					Y
DC208	NHC		F	62		N	N		N	N	N		AL	AL	N	W	N	N		Y	N
DC131	NHC		F	60		N	N		N	N	N		AL	AL	N	W	N	N		N	N
DC123	NHC	DP404	F	75		N	N		N	N	N			AL	N	W	N	N		N	N
DP164	PD		M	63	58	N	N		N	N	Y	5	NE	AL	N	W	N	N		N	N
DP038	PD		M	73	68	N	N			N	N		NY	AL	N	W	N			N	N
DP503	PD		F	58	48	N	N		N	N	Y but around or after onset	5	AL	AL	N	W	N	N		N	Y
DP148	PD		F	84	71	N	N		N	N	Y	4	AL	AL	N	W	N	N		Y	Y
SP0226	PD		F	61	59		Y	17						AL	N	W		N		N	N
SP0038M47	PD		M	68	65		N							AL	N	W				N	N
SP0080	PD		F	75	71		N							GA	N	W		N		N	Y
DP013	PD		M	86	80	N	N		N	N	N		AL	AL	N	W	N	Y	first	N	N
SP0066	PD		M	62	56		N							MS	N	W		Y	first	Y	Y
DP320	PD	DC109	F	66	50	N	N		N	N	Y		MO	AL	N	W	N	Y	second	N	
SP0021M44	PD		M	72	65		Y after onset	70						AL	N	W		N		Y	N
DP427	PD		F	65	64	N	N			N			AL	AL	N	W	N	Y	first	N	N
SC0561	NHC		M	70			N		N	N	N			AL	N	W	N	N		N	N
DP092	PD		F	72	68	Y	N		N	N	N		PA	AL	N	W	N			N	N
DC039	NHC	DP090	F	77		N	N		N	N	N		KY	AL	N	W	N	N		N	N
SP1091	PD		M	73	70		N		Y	N	Y				N	W	N	N		N	Y
DC194	NHC		F	68		N	N		N	N	N		AL	AL	N	W	N	N		Y	N
DP194	PD		M	77	70	N	Y	10	Y	N	N		IN	MS	N	W	N	Y	first	N	N
SP1025	PD		M	55	48		N		N	N	N			AL	N	W	N	Y	second	Y	N
DP367	PD		M	77	75	N	N		N	N	N		AL	AL	N	W	N	Y	first	N	N
SP0100	PD		M	59	55		N							AL	N	W		N		N	N
SC0530	NHC		M	61			Y	17	N	N	N				N	W	N	N		N	N
DP207	PD	DC069	F	63	25	N	Y after onset	62	N	N	N		FL	MS	N	W	N	Y	first	N	N
SC0635	NHC		F	72			Y	56	N	N	N			AL	N	W	N	N		Y	Y
SP0951	PD		M	56	52		N		N	N	N			AL	N	W	N	Y	third+	N	N
DC187	NHC		F	59		N	N		N	N	N		MS	MS	N	W	N	N		N	N

DC255	NHC		F	61		N	N		N	N	N		OH	AL	N	W	N	Y	first	N	N
SP0170	PD		M	71	70		N							AL	N	W		Y	first	N	N
DC159	NHC		F	56		N	N			N	N		AL	AL	N	W	N	Y	first	N	N
SC0079	NHC		F	75			N							AL	N	W		N		N	N
SP0275	PD		M	58	50				Y	N	N			AL	N	W	N	N		N	Y
SP0621	PD		M	67	61		N		N	N	N				N	W	N	N		Y	Y
SP0053	PD		M	64	53		N							AL	N	W		Y	first	Y	Y
SP0101	PD		M	40	27		N							AL	N	W		Y	third+	N	N
DP269	PD	DC090	M	66	55	Y	N		N	N	N		AL	AL	N	W	N	N		N	N
DP546	PD		M	71	65	N	N		N	N	N		AL	AL	N	W	N	N		N	Y
DP490	PD		F	63	55		N		N	N	N			AL	N	W				Y	N
DC006	NHC	DP009	M	37		N	N		N	N	N		AL	AL	N	W	N	N		N	N
DC090	NHC	DP269	F	65		N	N		N	N	N		AL	AL	N	W	N	N		N	Y
DC175	NHC		F	71		N	Y	13	N	N	N		AL	AL	N	W	N	N		N	Y
SP0235	PD		M	73	55		N							AL	N	W		N		N	N
SC0599	NHC		M	81			N		N	N	N			AL	N	W	Y	N		N	N
SC0019F50	NHC		F	65			N							AL	N	W		N		N	N
SP0633	PD		M	61	59		Y	16	Y	N	Y			AL	N	W	N			N	Y
SP1031	PD		M	75	74		Y	21	N	N	N			AL	N	W	N	Y	first	N	Y
SP0709	PD		M	59	55		N		Y	N	N			AL	N	W	N	N		N	N
SC0572	NHC		M	57			Y	14	Y	N	N			TN	N	W	N	N		N	N
SC0136	NHC		F	56			N							AL	N	W		N		N	N
DC009	NHC		F	73		N	N		N	N	N		AL	AL	N	W	N	N		N	N
DP497	PD		F	71	55	N	N		N	N	Y	70	GA	GA	N	W	N	Y	first	N	Y
SC0037	NHC		M	66			N							MS	N	W		N		N	N
DP632	PD	DC294	F	65	54	N	Y	22	N	N	N		GA	GA	N	W	N	Y	second	Y	Y
DP179	PD		M	77	66	N	Y	18	Y	N	N		IN	MS	N	W	N			N	N
DC064	NHC	DP188	F	54		N	N		N	N	N		TN	AL	N	W	N	N		N	N
SP0196	PD		M	68	62		N							AL	N	W		N		N	Y
DP155	PD		F	77	62	Y	N		N	N	N		MS	MS	N	W	N	N		N	Y
DP412	PD		F	50	33	Y	N			N	N		AL	AL	N	W	N	Y	second	Y	Y
DP600	PD		F	81	70	N	N		N	N	Y	10	IL	AL	N	W	N	Y	first	N	N
DP302	PD		F	77	64	N	N		N	N	N		MD	AL	N	W	N	N		N	N

DP159	PD		F	70	57	N	N		N	N	N		KY	AL	N	W	N	Y	second	N	N
DC107	NHC	DP309	F	54		N	N		N	N	N		AL	AL	N	W	N	N		N	N
SC0632	NHC		M	67			N		N	N				AL	N	W	N	N		N	N
DC032	NHC	DP065	F	62		N	N		N	N	N		AL	AL	N	W	N	N		Y	N
DC027	NHC	DP054	F	68		N	N			N	N		AL	AL	N	W	N	N		N	Y
DP018	PD	DC010	M	79	72	N	N		N	N	N		AL	AL	N	W	N	Y	first	N	Y
DP252	PD	DC085	M	74	65	Y	N		N	N	N		IN	AL	N	W	N	N		Y	Y
DP284	PD		M	55	45	N	Y	16	Y	N	N		AL	AL	N	W	N			N	Y
DP165	PD		M	61	52	Y	N		N	N	Y	23	MS	AL	N	W	N	Y	first	N	N
DP074	PD		M	74	63	N	N		N	N			Not US	AL	N	A	N	N		N	N
DP452	PD		F	66	63	N	N			N	N			AL		W				N	N
DP135	PD	DC054	M	79	65	Y	N		N	N	N		GA	GA	N	W	N	N		N	Y
SC0614	NHC		F	70			N		N	N	N			AL	N	W	N	N		N	N
DP007	PD	DC005	M	68	63	Y	N			N	N		AL	AL	N	W	N	N		Y	N
DP287	PD	DC096	F	66	62	N	N		N	N	Y	15	NY	AL		W	N	Y	second	N	N
SC0102	NHC		F	74			N							AL	N	W		N		N	N
DP281	PD		M	85	77	N	N		N	N	Y	7	AL	AL	N	W	N	Y	second	Y	Y
DP649	PD	DC317	M	67	50		Y	13	N	N	Y			AL	N	W	N	N		N	N
SP0137	PD		M	73	60		N							AL	N	W		N		N	N
DP223	PD	DC072	F	83	82	N			N	N	N		AL	AL	N	W	N	N		N	Y
DP277	PD	DC092	M	64	61	N	Y	5	N	N	N		Not US	AL	N	W	N	Y	second	N	Y
SP0332	PD		F	73	70		N		N	N	N			AL	N	W	N	N		N	N
DC007	NHC		F	60		N	N		N	N	N		AL	AL	N	W	N	N		N	Y
SP0034M64	PD		M	51	51		Y	16						AL	N	W		N		N	N
DP119	PD		M	84	80	Y	N		N	N	N		CA	AL	N	W	N	N		Y	N
DP486	PD		M	64	45	N	N		N		Y but around or after onset	2		AL		W				N	
DC265	NHC	DP570	F	55		N	Y	17	N	N	N		AL	AL	N	W	N	N		N	N
DP350	PD		M	69	58	N	N			N			TX	MS	N	W	N	N		N	N
DC097	NHC		F	67		N	N		N	N	N		CA	AL	N	W	N	Y	second	N	N
SP0005	PD		M	49	41		N							AL	N	W		Y	first	N	Y

DP116	PD		F	79	78	N	N		N	N	N		GA	FL	N	W	N	Y	second	N	Y
SP1004	PD		F	81	60		N		N	N	Y			AL	N	W		Y	second	N	N
SP0086	PD		M	73	57		N							AL	N	W		N		N	Y
SP0207	PD		M	50	29		N							AL		W		N		N	Y
DC186	NHC		F	65		N	N		N	N	N		AL	AL	N	W	N	N		N	N
DP172	PD	DC061	M	73	58	N	N		N	Y	N		AL	AL	N	W	N	N		N	Y
DP267	PD		F	73	64.5	N	N		N	N	Y	3	CA	AL	N	W	N	Y	first	Y	N
DP544	PD		M	65	62	N	N		Y	N	N		AL	AL	N	W	N	Y	third+	Y	N
DP040	PD		F	66	57	N	N		N	N	Y	6	AL	AL	N	W	N	N		N	Y
SC0099	NHC		F	68			N							SC	N	W		Y	third+	N	N
DC318	NHC		M	60			N		N	N	Y			AL	N	W	N	N		N	N
SC0126	NHC		F	64			N							AL	N	W		N		N	N
SP0975	PD		F	64	60		Y	32	N	N	N			AL	N	W	N	N		N	N
DP381	PD		M	79	77	N	Y	17	N	N	Y	6	AL	AL	N	W	N			N	Y
DP183	PD		F	63	59	N	N		N	N	N		AL	AL	N	M	N	Y	third+	Y	N
SP1072	PD		F	59	56		N		N	N	N			AL	N	W	N	N		N	N
SP0148	PD		M	59	49		N							AL	N	W		N		N	N
DP221	PD		F	66	62	N	N			N	N		NY	AL	N	W	N	Y	first	N	N
DP591	PD		M	65	52	N	Y	7	Y	N	Y	32	AL	AL	N	W	N	Y	first	N	N
DP073	PD	DC037	M	63	54	N	N		Y	N	N		AL	AL	N	W	N	Y	second	N	N
DP406	PD		F	65	50	N	N		N	N	N		AL	AL	N	W	N	N		N	Y
DP152	PD		F	80	79	N	N		N	N	N		PA	AL	N	W	N	N		Y	Y
SC0624	NHC		M	79			N		N	N	N			AL	N	W	N	N		Y	Y
SP0181	PD		M	68	43		Y	11.5						AL	N	W		N		N	Y
SP0085	PD		F	69	64		N							AL	N	W		Y	second	N	N
DP236	PD		F	71	67	N	Y after onset	68	N	N	N		AL	AL	N	W	N	Y	first	Y	N
SP0030M37	PD		M	78	70		N							AL	N	W		N		Y	Y
SC0059	NHC		F	57			N							AL	N	W		Y	third+	N	N
DP400	PD		M	63	56	N	Y	50	N	N	N		IL	AL	N	W	N	N		N	N
DP420	PD		F	60	45	N	N			N	Y	27	AL	AL	N	W	N	N		N	Y
SC0058	NHC		F	64			N							AL	N	W		N		N	Y
DC269	NHC		F	54		N	N		N	N	N		FL	FL	N	W	N	Y	second	N	N
DC287	NHC	DP615	M	66		N	N		N	N	N		NJ	FL	N	W	N	N		N	N

SP0216	PD		F	69	64		Y after onset	69						GA	N	W		N		N	N
DC185	NHC		F	59		N	N		N	N	N		AL	AL	N	W	N	Y	second	N	N
DP589	PD		M	51	40	N	N		Y	Y	Y	5	AL	AL	N	B	N	N		N	N
SP0071	PD		F	75	60		N							AL	N	W		Y	second	N	N
DP150	PD		F	63	52	N	N			N	N		AL	AL	N	W	N	Y	second	Y	Y
DP070	PD		F	72	59	N	N		N	N	N			AL	N	W	N	N		N	Y
SP0117	PD		M	74	72		N							AL	N	W		N		N	Y
DP447	PD		M	79	66	N	N		N	N	N		LA	AL	N	W	N	N		Y	Y
SP1045	PD		M	59	53		N		N	N	Y			AL	N	W	N	Y	first	N	N
SP1048	PD		M	46	44		N		N	N	N			AL	N	W	N	N		N	N
DP046	PD		F	55	48	Y	N			N			FL	AL	N	W	N	N		N	N
DC257	NHC		F	72		N	Y	30	N	N	N		WV	AL	N	W	N	N		N	N
DP001	PD		M	69	62	N	Y	16	N	N	N		AL	AL	N	W	N	N		Y	N
SC0145	NHC		M	59			N		N	N	N			AL	N	W	N	N		N	Y
SP1073	PD		F	84	68		Y	53	N	N	N			MS	N	W	N	N		N	Y
DP331	PD		M	78	74	N	Y	15	Y	N	N		AL	AL	N	M	N	N		Y	N
SC0531	NHC		M	85			N		N	N	N				N	W	N	N		N	N
SC0148	NHC		F	74			N		N	N	N			AL	N	W	Y	N		N	N
SC0119	NHC		F	83			N							AL	N	W		Y	first	N	N
DP518	PD		F	73	63	N	N		N	N	N		AL	AL	N	W	N	N		N	Y
SP0946	PD		F	69	60		N		N	N	N			AL	N	W	N	Y	second	N	N
DP273	PD		F	61	56	N	N			N	Y	18	AL	AL	N	W	N	Y	first	N	N
DP079	PD		M	70	59	N	N		N	N	N		GA	GA	N	W	N	N		N	N
DC189	NHC		M	66		N	N			N	N		TN	TN	N	W	N	N		N	N
DP580	PD		M	68	67	N	N		N	N	N		AL	AL	N	W	N	N		Y	N
DC310	NHC		M	72		N	N		N	N	N		PA	AL	N	W	N	N		N	N
SP0211	PD		M	80	60		N							GA	N	W		N		Y	Y
SC0600	NHC		F	80			Y	11	N	N	N			AL	N	W	Y	N		N	N
SP0188	PD		M	69	58		Y	58						SC	N	W		N		N	N
SP0959	PD		F	60	57		N		N	N	N			AL	N	W	N	N		N	Y
DP553	PD		M	72	50	Y	N		N	N	N		GA	TN	N	W	N	N		N	Y
DP138	PD	DC057	M	68	67	N	N		N	N	N		AL	AL	N	W	N	N		N	N
DP255	PD	DC082	M	65	62	N	N		N	N	Y	28	AL	AL	N	W	N	N		Y	N

SP0027F46	PD		F	69	50		N							AL	N	W		Y	second	N	Y
SP0093	PD		M	62	46		N							AL	N	B		Y	third+	N	N
SP0164	PD		M	69	52		N		N	N	Y			MS	N	W	Y	N		N	Y
DC235	NHC		F	59		N	Y	22	N	N	N		MS	AL	N	W	N	Y	second	N	
DP360	PD		M	62	46	N	N		N	N	Y		AL	AL	N	W	N	Y	third+	N	
DP124	PD		F	77	76	N	N		N	N	Y	18	MS	MS	N	W	N	Y	second	N	Y
DC172	NHC		F	68		N	N		N	N	N		GA	AL	N	W	N	N		N	N
DC114	NHC	DP353	M	59		N	N		N	N	N		PA	AL	N	W	N	N		N	N
SP0940	PD		F	70	67		N			N				AL	N	W	N	N		N	
DC110	NHC	DP334	F	64		N	N		N	N	N		AL	AL	N	W	N	N		N	N
SP0017	PD		M	68	60		N							MS	N	W		Y	first	N	Y
DP037	PD		F	71	61	N	N		N	N	N			AL		W				N	Y
SP0138	PD		M	84	69		N							AL	N	W		Y	first	Y	Y
DP084	PD		F	84	79	N	N		N	N	Y		AL	AL	N	W	N	N		N	N
DC230	NHC		F	71		N	N		N	N	N		AL	AL	N	W	N	N		N	N
SP0982	PD		F	65	54		N		N	N				AL	N	W	N	Y	second	Y	Y
SP0040M42	PD		M	73	72		N							AL	N	W		N		N	Y
SC0259	NHC		F	71			N		N	N	N			AL	N	W	N	N		N	Y
SC0566	NHC		M	59			N		N	N	N			AL	N	W	N	N		N	N
DP226	PD		M	53	36	N	N		N	N	N		Not US	AL	N	A	N	N		N	
SP0136	PD		F	71	42		Y after onset	60						AL	N	W		Y	first	Y	Y
DC263	NHC	DP564	F	60		N	N		N	N	N		AL	AL	N	W	N	N		N	N
DP638	PD		M	65	53	N	N		N	N	N			AL	N	W				N	
DP346	PD	DC113	M	77	66	N	N		N	N	Y		AL	AL	N	W	N	N		N	Y
DP334	PD	DC110	M	66	53	Y	N		Y	N			AL	AL	N	W	N	N		N	Y
DC314	NHC		F	68		N	N		N	N	Y	10	AL	AL	N	W	N	N		N	N
DP530	PD	DC199	M	69	62	N	N		N	N	N		MS	MS	N	W	N	N		N	N
DC248	NHC		F	50		N	N		N	N	N		AL	AL	N	W	N	Y	first	N	Y
DP375	PD		F	77	70	N	N		N	N	Y	4	AL	AL	N	W	Y	Y	first	Y	N
SC0038	NHC		M	77			N							GA	N	W		N		N	N
DP562	PD		M	60	58	N	N		Y	N	N		FL	FL	N	W	N	N		Y	N
SP0192	PD		F	67	64		Y	6						AL	N	W		N		Y	Y
SC0056	NHC		M	79			N							NC	N	W		N		N	N

DP156	PD		M	59	53	N	Y	17	Y	N	Y	16	AL	AL	N	W	N	Y	second		Y
DP300	PD	DC102	M	71	53	N	Y	28	N	N	Y	30	AL	AL	N	W	N	N		N	Y
DP006	PD	DC003	M	62	42	N	N		N	N	Y		TN	AL	N	W	N	N		N	N
SP0231	PD		F	42	37		N							AL	N	W				N	N
SP0968	PD		F	71	68		N		N	N	N			NC	N	W	Y	Y	first	N	N
DC298	NHC		F	56		N	N		N	N	N		AL	AL	N	W	N	Y	second	N	N
SP0115	PD		M	66	53		N							AL	N	W		Y	first	N	N
SC0094	NHC		M	73			N							AL	N	W		N		N	N
SP0081	PD		F	54	47		N							AL	N	W		Y	second	N	N
DC022	NHC	DP047	F	68		N	N			N	N		AL	AL	N	W	N	N		N	N
SP0122	PD		M	75	71		N							MS	N	W		N		Y	N
DP389	PD		M	66	64	N	N		N	N	N		KY	AL	N	W	N	N		N	N
DP310	PD	DC108	M	69	50	N	N		N	N	N		AL	AL	N	W	N	Y	first	N	Y
DP480	PD		M	78	67	N	Y	11	Y	N	N		VA	AL	N	W	N	Y	first	Y	N
SP0119	PD		M	61	59		Y	12						AL	N	W		N		N	Y
SC0591	NHC		F	51			N		N	N	N			AL	N	W	N	N		N	N
SC0607	NHC		F	69			N		N	N	N			AL	N	W	N	N		N	N
SP0179	PD		M	76	70		N							AL	N	W		N		N	N
DP578	PD		F	78	74	N	N		N	N	Y		AL	AL	N	W	N	N		N	N
DP467	PD	DC147	M	79	69	N	N		N	N	N		LA	AL	N	W	N	N		N	
DP213	PD		M	55	35	N	N		N	N	Y	2	AL	AL	N	W	N	Y	first	N	Y
SC0072	NHC		F	57			N							AL	N	W		N		N	N
DC297	NHC		F	67		N	N			N	N		AL	AL	N	W	N	N		N	N
SP0430	PD		F	77	62		N		N	N	Y			AL	Y	W		Y	second	Y	N
DP605	PD		F	59	54	Y	N		N	N	Y	10	AL	AL	N	W	Y	N		N	Y
SC0529	NHC		F	68			N		N	N	Y			AL	N	W	N	N		Y	Y
DP086	PD		F	64	50	Y	N		N		N			AL	N	B	N	N		N	N
DC198	NHC	DP527	F	61		N	N		N	N	N		IL	AL	N	W	N	N		N	Y
DP537	PD		M	67	52	N	N		N	N	N		LA	AL	N	W	N	Y	third+	N	N
SP0254	PD		M	79	71		N		N	N	N			Al	N	W	Y	Y		N	Y
DP285	PD	DC095	F	62	60	N	N		N	N	N			AL		W				Y	Y
DP322	PD		M	77	62	N	N				Y	60	AL	AL	N	W	N	N		N	Y
DP493	PD		F	67	64	N	Y	12	N	N	Y	2	SC	GA	Y	W	N	Y	second	Y	N

DP443	PD		F	62	48	N	N		N	N	Y	25	AL	AL	N	W		N		N	N
DP293	PD		M	68	62	N	Y	14	Y	N	Y but around or after onset		AL	AL	N	W	N	Y	first	Y	N
DC153	NHC	DP491	F	71		N			N	N			AL	AL	N	W	N	Y	first	Y	
DC004	NHC	DP014	F	72		N	N		N	N	N		AL	AL	N	W	N	N		N	N
DP501	PD	DC155	M	60	58	N	Y	18	N	N	N		AL	AL	N	W	N	Y	second	Y	N
DP451	PD		M	78	73	N	Y	7.5	N	N	N		AL	AL	N	W	N	N		Y	Y
DP153	PD		M	85	62	Y	Y	55	N	N	Y	17	AL	AL	N	W	N	N		Y	Y
DP186	PD	DC063	M	79	72	N	Y	20	N	N	N		WY	AL	N	W	N	N		N	N
DP347	PD		M	76	60	Y	N		N	N	N		VT	AL	N	W	N	N		N	Y
SC0097	NHC		F	76			N							FL	N	W		N		N	N
SP1024	PD		M	79	70		N		N	N	N			MS	N	W	N	Y	first	N	N
DP275	PD		F	80	71	N	N		N	N	N		AL	AL	N	W	N	N		N	N
DP529	PD		M	69	67	N	N		N	N	N		AL	AL	N	W	N	N		N	N
SP1108	PD		F	70	64		N		N	N	Y			AL	N	W	N	N		Y	Y
DC001	NHC	DP004	M	72		N	Y	19	N	Y	N		LA	AL	N	W	N	N		N	N
SP0229	PD		F	61	50		N							AL	N	W	Y	Y	first	N	Y
DC100	NHC	DP296	M	73		N	N		N	N	N		AL	AL	N	W	N	N		N	N
DC266	NHC		F	67		N	N		N	N	N		ME	AL	N	W	N	N		N	Y
DP568	PD		F	68	60	N	N		N	N	Y	18	AL	AL	N	W	N	N		N	N
DP489	PD		F	61	50		N		N	Y	N		AL	AL	N	B	N	N		N	N
DC066	NHC	DP192	F	72		N	N		N	N	N		DC	AL	N	W	N	N		N	N
DP048	PD		F	76	54	Y	N		N		Y	75	AL	AL	N	W	N	Y	first	N	Y
DP436	PD	DC137	F	65	55	N	N		N	N	Y	65	AL	AL	N	W	N	Y	first	N	N
DP017	PD		M	56	47	N	Y	6	Y	N	N		AL	AL	N	W	N	N		Y	N
DP504	PD		M	56	50	N	N		Y	N	N		AL	AL	N	W	N	N		Y	N
SC0617	NHC		M	53			Y	15	Y	N	N			AL	N	W	N	N		N	N
DP049	PD	DC023	M	75	71	Y	N			N	Y	18	TX	MS	N	W	N	Y	second	N	Y
SP1090	PD		F	71	65		N		N	N	Y			AL	N	W	N	N		N	Y
SC0067	NHC		F	74			N							AL	N	W		Y	second	N	Y
DP107	PD	DC128	M	69	40	N	Y	28	N	N	Y	2	LA	AL	N	W	N	Y	second	N	Y
SP0109	PD		M	70	65		N							AL	N	W		N		N	N

DP385	PD	DC117	F	74	62	N	N		N	N	N		MS	AL	N	W	N	Y	first	Y	N
SP0099	PD		M	56	45		Y	17						AL	N	W		N		N	N
SC0557	NHC		F	54			N		N	N	Y			MS	N	W	N	N		N	N
DC017	NHC	DP028	F	68		N	Y	20	N	N	N		AL	AL	N	W	N	Y	first	N	N
DC243	NHC		F	63		N	N		N	N	N		TN	AL	N	W	N	N		N	N
SC0535	NHC		F	50			Y	10	N	N	N				N	W	N	N		Y	Y
SP0074	PD		M	74	66		N							AL	N	W		Y	first	N	N
DP477	PD		M	73	67	N	N		N	N	N		AL	AL	N	W	N	Y	first	N	Y
DP258	PD		M	81	80	N	N		N	N	N		VA	AL	N	W	N	N		N	N
DP091	PD		F	69	57	Y	N		N	N	Y		AL	AL	N	W	N	Y	second	N	N
DP470	PD		M	73	71	N	N		N	N	N		Not US	AL	N	W	N			Y	N
DP610	PD		M	71	68	N	N		N	N	N		AL	AL	N	W	N	N		N	N
DC204	NHC		M	65		N	N		N	N	N		AL	AL	N	B	N	N		N	Y
DP304	PD	DC103	F	73	61	N	Y	14	N	N	N		TN	TN	N	W	N	Y	first	Y	N
DP550	PD		M	77	74	N	N		N	N	N		AL	AL	N	W	N	N		N	N
DC145	NHC		F	41		N	N		N	N	N		AL	AL	N	W	N	N		N	N
DP053	PD	DC026	M	73	59	N	N		N	N	N		AL	AL	N	W	N	Y	first	N	Y
DC010	NHC	DP018	F	75		N	N			N	N		AL	AL	N	W	N	Y	third+	N	N
SP0127	PD		M	64	55		Y							AL	N	W		N		Y	N
DP239	PD		M	66	42	N	N		N	N	N		AL	AL	N	W	N	Y	second	N	N
SP1103	PD		M	74	72		N		Y	N				AL	N	W	N	N		N	Y
DC252	NHC		M	70		N	N		N	N	N		IA	AL	N	W	N	N		Y	N
DP472	PD		M	82	75	N	N		N	N	N		AL	AL	N	W	N	Y	first	N	N
SP0069	PD		F	57	45		N							AL	N	W		N		Y	
SP0987	PD		M	64	50		Y	16	Y	N	Y			AL	N	W	N	Y	first	N	Y
SC0583	NHC		F	74			N		N	N	N			AL	N	W	N	N		N	N
SP0142	PD		F	55	50		Y	34						AL	N	W		Y	second	N	N
SP0022M52	PD		M	63	59		N							AL	N	W		N		N	N
SP0023F57	PD		F	58	57		Y	8.5						AL	N	W		Y	first	N	Y
DP463	PD		F	75	63	N	N		N	N	N			OK	N	W	N	N		Y	Y
DP514	PD	DC171	M	60	52	N	N		Y	N	N		NY	AL	N	W	N	N		N	N
DC147	NHC	DP467	F	74		N	N		N	N	N		LA	AL	N	W	N	Y	second	N	N
DP030	PD		M	72	66	N	N		N	Y	N		AL	AL	N	W	N	N		N	N

SP0966	PD		F	76	68		Y	65	N	N	N			AL	N	W	N	Y	third+	N	N
SP0082	PD		F	75	74		Y	5						AL	N	W		Y	second	N	N
DC096	NHC	DP287	M	76		N	N			Y			MA	AL	N	W	Y	N		N	N
SP0274	PD		M	60	56		N		Y	N	Y				N	W	N	Y	first	N	N
SP0126	PD		F	65	56		N							GA	N	W		N		Y	N
DP380	PD		M	58	45	N	N		N	N	N		TX	AL	N	W	N	N		N	N
DP004	PD	DC001	F	73	60	N	N			N	N		Not US	AL	N	W	N	Y	second	N	N
SP1082	PD		M	72	64		N		N	N	N			AL	N	W	N	Y	second	N	N
SC0554	NHC		M	44			N		Y	N	N			AL	N	W	N	N		Y	N
SC0585	NHC		F	76			N		N	N	N				N	W	N	N		N	N
DP499	PD		M	69	62	Y	N		N		Y	18	VA	AL	N	B	N	N		N	N
DC292	NHC	DP625	F	79		N	N		N	N	N			AL		W		N		N	Y
DC205	NHC		F	70		N	N		N	N	N			AL		W				N	N
DP621	PD		F	71	62	N	N		N	N	Y	20	MI	AL	N	W	N	N		N	Y
DP637	PD		F	66	40	Y	N		N	N	N		AL	AL	N	W	N	N		Y	Y
SC0569	NHC		F	69			N		N	N	N			AL	N	W	N	N		Y	N
SC0088	NHC		F	71			N							AL	N	W		N		N	N
SP0135	PD		F	63	52		N							AL	N	W		N		Y	N
SP0153	PD		M	63	48		N							AL	N	W		N		N	N
SP0972	PD		M	75	69		N		N	N	N			MS	N	W	N	N		N	N
SP0057	PD		M	63	48		N							AL	N	W		N		N	N
SP0058	PD		M	76	64		N							MS	N	W		N		N	Y
DP098	PD		F	70	65	N	N		N	N	N		AL	AL	N	W	N	N		N	N
SC0497	NHC		F	60			N		N	N	N				N	W	N	N		N	N
DP002	PD		M	56	42	N	N		N	N	N		AL	AL	N	W	N	Y	second	N	N
SP0948	PD		F	75	71		Y	71	N	N	Y			AL	N	W	N	Y	second	N	N
DC317	NHC	DP649	F	64			N		N	N	Y			AL	N	W	N	N		N	N
DP601	PD		M	67	56	N	N		N	N	N		MN	AL	N	W	N	Y	second	N	Y
DC193	NHC		F	73		N	N		N	N	N		MS	AL	N	W	N	Y	second	N	N
DP567	PD		M	78	75	N	N		N	N	N		MO	MS	N	W	N	N		N	N
SP1092	PD		M	74	67		Y	11	Y	N	Y			MS	N	W	N	N		Y	Y
SC0150	NHC		F	59			N		N	N	N			AL	N	W	N	N		N	N
DC212	NHC		M	74		N	N		N	N	N		AL	AL	N	W	N	Y	second	N	N

DP289	PD	DC098	M	80	59	N	N			Y	Y	4	NE	AL	N	W	N	N		N	Y
SP0252	PD		M	56	52		N							AL	N	W		N		N	Y
DP348	PD		M	72	67	Y	N			N	N		CA	AL	N	M	N	N		N	N
SP0251	PD		M	77	64		N							AL	N	W		N		N	N
SC0016F52	NHC		F	63			Y	7						AL	N	W		Y	second	N	N
DP623	PD		M	70	67	N	N		N	N	N		AL	AL	N	W	N	N			
SP0236	PD		F	79	73		N							AL	N	W		N		N	N
DC141	NHC	DP449	M	76		N	N		N	N	N		Not US	AL	N	W	N	N		N	N
DP243	PD		F	71	57	N	N		N	N	N		MS	MS	N	W	N	Y	first	Y	Y
DP198	PD	DC067	F	66	63	N	Y	43	N	N	Y	1	AL	AL	N	W	N	N		N	N
SP0047M47	PD		M	68	62		N							TN	N	W		Y	third+	N	N
SP0018	PD		M	83	78		N							AL	N	W		N		N	N
DP364	PD		M	75	72	N	Y	10	N	N	N		AL	AL	Y	W	N	Y	first	N	Y
DP005	PD		F	65	60	N	N		N	N	Y	7	MS	MS	N	W	N	Y	first	Y	Y
DP335	PD		M	58	57	N	N		N	N	Y		OH	AL	N	W	N	Y	second	N	N
DP112	PD		M	71	66	N	N			N	N		TX	AL	N	W	N	N		N	N
DP249	PD	DC078	F	77	61	N	N		N	N	Y	26	AL	AL	N	W	N	Y	first	N	Y
SC0011F45	NHC		F	70			N							AL	N	W		N		N	N
DP296	PD	DC100	F	72	69	N	N		N	N			AL	AL	N	W	N	Y	first	N	N
SC0546	NHC		M	59			N		N	N	N			AL	N	W	N	N		N	N
DP351	PD		F	57	42	N	N		N		N			AL	N	W	N	N		N	Y
SP0129	PD		M	54	38		Y	18						AL	N	W		Y	second	N	Y
DP021	PD		M	77	64	N	N			N	Y	25	MS	MS	N	W	N	Y	second	N	Y
DC160	NHC	DP506	M	77		N	N				Y	55	KY	AL	N	W	N	N		N	N
DP368	PD		M	71	68	N	N		N	N	Y	18	WI	AL	N	W	N	N		N	N
SP0993	PD		F	54	36		N		N	N	N			AL	N	W	N	N		N	Y
DC058	NHC	DP139	F	69		N	N		N	N	N		OH	AL	N	W	N	Y	second	N	N
DC176	NHC		F	47		N	Y	25	N	N	N		MS	AL	N	W	N	N		Y	N
DP539	PD		F	65	55	Y	N		N	N				AL	N	W				N	Y
DP240	PD		M	72	51	N	Y	49	N	N	N		KY	TN	N	W	N	N		Y	Y
SP0102	PD		F	85	65		Y	18						FL	N	W		Y	first	Y	Y
DC035	NHC		M	64		N	Y	23	Y	N	N		TN	TN	N	W	N	N		N	N
DC087	NHC	DP262	M	67		N	N		N	N	Y	12	GA	FL	N	W	N	N		Y	N

DP396	PD		F	75	63	N	N		N	N	Y	17	AL	AL	N	W	N	N		Y	Y
SC0006	NHC		M	64			N							AL	N	W		N		Y	N
SC0057	NHC		F	68			N							AL	N	W		N		N	N
DP587	PD		M	49	44	N	N				N		AL	AL		W					
DC139	NHC		F	47		N	N		N	N	N		KY	AL	N	W	N	N		N	N
SP0219	PD		M	64	49		N							GA	N	W		N		N	N
DC021	NHC	DP045	F	68		N	N		N	N	Y	67	MS	MS	N	W	N	Y	first	N	N
SP1007	PD		F	57	52		N		N	N	N			AL	N	W	N	N		N	Y
DC121	NHC		M	66		N	N		N	N	N		AL	AL	N	W	N	N		N	N
DP624	PD		F	74	56	N	N		N	N	N		AL	AL	N	W	N	N		Y	Y
DP041	PD		F	64	61	N	Y	44	N	N	Y	5	MS	AL	N	W	N	N		Y	Y
DC201	NHC		M	77		N	N		N	N	N		GA	AL	N	W	N	N		N	N
DC276	NHC		F	72		N	N		N	N	N		NY	AL	N	W	N	N		N	N
DP292	PD	DC174	F	60	55	N	N		N	N	N		IA	AL	N	W	N	Y	third+	N	N
SC0015F46	NHC		F	69			N							AL	N	W		N		N	N
DP276	PD		M	81	65	N	N		N	N	Y	14	AL	AL	N	W	N	N		Y	Y
SP0942	PD		F	66	64		N		N	N	N			AL	N	W	N	Y	second	N	N
DP055	PD		M	61	48	N	N		N	N	N		GA	AL	N	W	N	Y	second	Y	Y
SC0029	NHC		F	87			N							AL	N	W		N		N	N
DP398	PD		F	40	37	N	N		N	N	N		AL	AL	N	W	N	N		N	N
SP1083	PD		F	77	76		Y	60	N	N	N			AL	N	W	N	N		N	N
DC234	NHC		F	63		N	N		N	N	N		AL	AL	N	W	N	N		Y	N
DC229	NHC		F	69		N	N		N	N	N		IA	AL	N	W	N	N		N	N
DC018	NHC	DP031	F	80		N	N		N	N	N		AL	AL	N	W	N	N		N	N
SC0047	NHC		F	72			N							AL	N	W		Y	first	N	Y
SP0962	PD		F	71	55		N		N	N	Y			AL	N	W	N	N		N	Y
DP072	PD		M	73	70	N	Y	53	N	N	N		PA	AL	N	W	N	N		N	N
DP340	PD		F	53	40	N	N		N	N	Y	10	AL	AL	N	W	N	Y	first	N	Y
SC0618	NHC		M	84			N		N	N	N				N	W	N	N		N	N
SC0597	NHC		F	63			N		N	N				GA	N	W	N	Y	first	N	N
DP439	PD		F	57	30	N	N		N	N	N		AL	MS	N	W	N	N		N	N
DC109	NHC	DP320	M	60		N	N		N	N	N		ME	AL	N	W	N	N		N	N
DP120	PD		M	67	55		N		N	N			AL	AL	N	W	N	Y	first	Y	N

SC0590	NHC		F	77			N		N	N	N			MS	N	W	N	N		N	N
SC0078	NHC		F	70			N							AL	N	W		Y	second	Y	N
DP333	PD	DC081	M	85	68	N	N		N		N		AL	AL	N	W	N	N		Y	Y
DC082	NHC	DP255	F	52		N	N		N	N	Y	51	AL	AL	N	W	N	N		N	N
DC108	NHC	DP310	F	66		N	Y	10	N	N	N		AL	AL	N	W	N	N		N	N
SP0133	PD		F	65	59		N							MS	N	W		Y	second	Y	N
SP0033F47	PD		F	68	66		N							AL	N	W		N		N	N
DP203	PD		M	77	64	N	N		Y	N	N		Not US	AL	N	W	N	N		N	N
DC071	NHC		M	73		N	N		N	N	N		IL	MS	N	W	N	N		N	N
SC0588	NHC		F	83			N		N	N	N			FL	N	W	N	N		N	N
SC0303	NHC		F	60			N		N	N	N				N	W	Y	N		Y	Y
DP356	PD		M	77	66	N	N		N	N	N		AL	AL	N	W	N	N		N	N
SP0240	PD		F	55	44		N							AL	N	W		Y	second	N	Y
SP0091	PD		M	83	78		N							AL	N	W		N		N	Y
SP0246	PD		M	59	34		N							AL	N	W		Y	first	N	Y
DP319	PD		M	70	66	N	N		N	N	Y	2	GA	AL	N	W	N	N		N	N
SP0162	PD		F	62	56		N							AL		W		N		N	Y
SP1064	PD		M	64	19		Y	16	N	N	Y				N	W	Y	Y	first	N	Y
SP1049	PD		M	70	55		N		Y	N	Y			AL	N	W	N	Y	first		
SP0218	PD		M	76	62		N							AL	N	W		Y	second	N	N
DC244	NHC		M	78		N	N		N	N	N		AL	AL	N	W	N	N		N	N
DC099	NHC	DP295	F	59		N	Y	16	N	N	N		NY	AL	Y	W	N	N		N	N
DC174	NHC	DP292	M	60		N	N		N	N	N		NE	AL	N	W	N	N		Y	N
DC068	NHC		F	61		N	N		N	N	N		FL	AL	N	W	N	N		N	N
DC044	NHC	DP100	F	67		N	N		N	N	N		HI	AL	N	W	N	N		N	N
DP494	PD		M	78	70	N	N		N	N	N			AL	N	W				N	Y
DP415	PD		F	72	56	N	N		N	N	N			AL	N	W				Y	Y
DP354	PD	DC115	F	62	53	N	N		N	N	N		VA	GA	N	W	N	N		N	N
DP464	PD		M	66	49	N	Y after onset	56	N	N	N		AL	AL	N	M	N	N		Y	Y
SC0030	NHC		F	74			N							AL	N	W		N		N	N
DP639	PD	DC312	M	72	65	N	N		N	N	N		AL	AL	N	W	N	Y	second	N	N
SC0587	NHC		F	64			N			N	N			AL	N	W	Y	Y	third+	N	N
SP0247	PD		M	59	41		N							AL	N	W		Y	second	Y	N

SP0413	PD		M	64	61		N		Y	N	N			AL	N	W	N	N		Y	N
SP0028F60	PD		F	55	54		N							AL	N	B		N		Y	N
SP1032	PD		M	69	64		N		N	N	N			AL	N	W	N	Y	second	N	
DC281	NHC		M	60		N	N		N	N	N		KY	FL	N	W	N	N		N	N
DP500	PD		F	70	60	N				N	Y	1	GA	AL	N	W	N	N		N	Y
SC0542	NHC		F	60			N		N	N	Y			AL	N	W	N	N		N	N
DP308	PD	DC106	F	63	54	Y	Y after onset	64	N	N	N		MS	MS	N	W	N	Y	second	N	Y
SP0297	PD		F	77	67		N		N	N	N				N	W	N	Y	first	N	N
SP0580	PD		M	70	63		N			N	Y				N	W	Y	N			Y
DP509	PD		F	71	58	N	N		N	N	Y	17	MS	MS	N	W	N	Y	first	N	N
DP376	PD		F	72	56	N	N		N	N	N		MS	MS	N	W	N			N	Y
DP641	PD		M	66	58	N	N		N	N	N		NC	SC	N	W	N	N		Y	Y
SP0140	PD		M	68	60		Y	10						AL	N	W		Y	second	N	Y
DP594	PD		M	82	75	N	N		N	N	N		VA	AL	N	W	N	N		N	N
DP054	PD	DC027	M	74	52	N	N		N	Y	N		AL	AL	N	W	N	N		N	Y
SC0080	NHC		F	51			N							MS	N	W		N		N	N
DP108	PD		F	77	75	N	N		N	N	Y	1	Not US	AL	N	W	N	Y	third+	N	N
DP450	PD		M	73	64	N								AL	N	W				Y	Y
SP0289	PD		M	63	51		N		Y	N	Y			AL	N	W	N	N		Y	N
SP1080	PD		M	78	71		N			N	N			AL	N	W	N	N			Y
DP613	PD	DC285	M	69	64	N	N		N	N	N		AL	AL	N	W	N	Y	first	N	N
SP0113	PD		F	80	66		N							NC	N	W				N	Y
SC0068	NHC		M	65			N							MS	N	W		N		N	Y
SP0144	PD		M	68	62		N							MS		W		N		N	
DP392	PD	DC118	M	71	60	N	N		N	N	N		AL	AL	N	W	N	N		N	Y
DP028	PD	DC017	M	73	66	N	N		N	N	N		AL	AL	N	W	N	N		Y	N
DP571	PD		M	75	50	N	N		N	N	Y		MS	MS	N	W	N	N		N	Y
DC092	NHC	DP277	F	64		N	N		N	N	N		AL	AL	N	W	N	N		N	Y
DP321	PD		F	72	61	N	N		N	N			MS	MS	N	W	N	Y	first	N	Y
DC247	NHC		M	71		N	N		N	N	N		AL	AL	N	B	N	N		N	N
SC0042	NHC		F	71			N							AL	N	W		N		N	N
DP039	PD		F	69	66	N	N		N	N	Y		VA	LA	N	W	N	Y	third+	Y	Y
SC0064	NHC		F	56			N							AL	N	W		N		N	N

SP1106	PD		M	81	36		N		N	N	N			AL	N	W	N	Y	first	N	Y
SC0598	NHC		F	77			N		N	N	N			AL	N	W	N	N		N	N
SP0131	PD		M	58	32.5		Y	32						AL	N	W		N		Y	Y
SP1079	PD		M	59	30		N		N	N	N			AL	N	W	N	N		N	N
SP0997	PD		F	58	48		N		N	N	N			MS	N	W	N	N		N	Y
DP473	PD		M	63	55	Y	N		N	N	Y	21	AL	AL	N	W	N	Y	first	N	N
DC300	NHC	DP151	F	71		N	N		N	N	N		AL	AL	N	W	N	N		N	N
DP547	PD	DC249	M	53	46	N	N		Y	N	N		OK	AL	N	W	N	N		N	N
SC0051	NHC		F	62			Y	20						FL	N	W		N		Y	N
DP422	PD		F	45	45	Y	N		N	N	N		AL	AL	N	W	N	N		N	Y
SP0157	PD		M	77	73		N							AL	N	W		N		N	Y
SP0281	PD		F	85	75		N		N	N	N			AL	N	W	N	Y	first	N	N
SP0146	PD		M	75	68		N							AL	N	W		N		N	Y
DC188	NHC		M	72		N	N				N		IL	AL	N	W	N	N		N	N
SP1033	PD		M	69	62		N		N	N	Y			AL	N	W	N	N		N	N
SP0063	PD		M	75	65		Y	16						AL	N	W		Y	second	N	Y
DP204	PD		F	54	52	N	N		N	N	N		AL	AL	N	W	N	N		N	N
SP1019	PD		M	72	69		N			N	N			AL	N	W		N		N	N
DP515	PD		M	72	67	N	Y	13	Y	Y	N		AL	AL	N	W	N	N		Y	N
DP523	PD		M	72	70	N	N		N	Y	N		AL	AL	N	W	N	Y	second	N	Y
DP541	PD		F	74	65	N	N			N	Y	73	IN	TN	N	W	N	N		N	Y
DP301	PD		M	74	60	N	N			N	N			AL	N	W	N	N		N	N
SC0086	NHC		F	64			N							GA	N	W		Y	second	N	N
DP508	PD		M	68	63	N	Y	16	N	N	N		AL	AL	N	W	N	Y	first	N	N
DC202	NHC		F	74		N	N		N	N	N		AL	AL	N	W	N	N		N	N
DP618	PD		M	75	71	N	Y	20	Y	N	Y	60	AL	AL	N	M	N	Y	first	N	N
DC034	NHC	DP071	M	81		N	N		N	N	N		AL	AL		W		N		Y	N
DC105	NHC	DP307	M	75		N	N		N				MS	AL	N	B	N	N		N	N
SP0141	PD		F	53	30		N							AL	N	W		Y	first	Y	N
DP008	PD		M	76	56	N	N		N	N	N		PA	AL	N	W		N		N	Y
DC282	NHC		F	70		N	N		N	N	N		VA	AL	N	W	Y	N		N	N
DP324	PD		M	70	65	N	N		N	N	N		AL	AL	N	W	N	N		Y	Y
SP0447	PD		M	69	56		Y		N	N	N			GA	N	W	N	Y	first	N	Y

DP234	PD		M	69	54	N	Y	32.5	N	N	Y	69	TN	TN	N	W	N	Y	first	N	N
SP1036	PD		M	66	49		N		N	N	N			GA	N	W	N	Y	first	N	N
SC0615	NHC		M	77			Y	16	N		Y			AL	N	W	N	N		N	Y
DP453	PD		F	75	72	N	N		N	N	N		AL	AL	N	W	N	Y	first	Y	Y
SP0603	PD		M	71	64		N		N	N	N			AL	N	W	N	N		N	Y
DC115	NHC	DP354	M	69		N	N		N	N	N		GA	GA	N	W	N	N		N	N
DP640	PD		M	74	69	N	N			N	Y	7	AL	AL	N	W	N	Y	third+	N	N
SP0284	PD		F	78	72.5		N		N	N	N			AL	N	W	N	N		N	N
DP090	PD	DC039	M	72	49	N	Y	11	Y		Y		KY	AL	N	W	N	Y	second		N
SP0096	PD		F	85	65		N							AL	N	W		Y	first	N	Y
SC0579	NHC		F	61			N		N	N	N			AL	N	W	N	N		N	N
SC0575	NHC		F	70			N		N	N	N			GA	N	W	N	Y	third+	N	N
DC200	NHC	DP532	F	65		N	N		N		Y	13	CA	FL	N	W	N	N		Y	N
SC0143	NHC		M	80			N		N	N	N			AL	N	W	N	N		N	N
DC095	NHC	DP285	M	70		N	N		N	N	N		MN	AL	N	W	N			N	N
DP114	PD		M	63	52	N	N		N	N	N		AL	AL	N	W	N	Y	first	N	N
DP405	PD	DC126	M	73	62	Y	Y	14	N	N	Y	52	AL	AL	N	W	N	Y	first	N	N
DC239	NHC		F	62		N	N		N	N	N		TN	AL	N	W	N	N		N	N
DC270	NHC	DP581	M	72		N	N		N	N	N		MS	MS	N	W	N	N		N	N
DP419	PD		M	68	67	N	N		N	N	N			AL	N	W	N	N		Y	N
SP0130	PD		F	73	63		N							AL	N	W		N		N	Y
SP0610	PD		F	67	65.5		N		N	N	N			AL	N	W	N	N		N	N
SC0026	NHC		F	72			N							AL	N	W		N		N	N
SC0249	NHC		M	54			N		N	N	N			AL	N	W	N	N		N	N
DP369	PD		M	64	58	N	Y	16	Y	N	N		AL	AL	N	W	N	N		N	N
DP307	PD	DC105	F	71	50		N		N	N	N			AL	N	B				N	
DC056	NHC	DP137	F	59		N	N		N	N	Y	16	TN	AL	N	W	N	Y	first	N	N
DC003	NHC	DP006	F	59		N	N		N	N	N		Not US	AL	N	A	N	N		N	N
DP332	PD		M	69	63	N	N		N	N	N		AL	AL	N	W	N	N		N	N
DC181	NHC		F	55		N	N			N	N		FL	AL	N	W	N	N		N	N
SP1054	PD		F	79	74		N		N	N	N			AL	N	W	N	N		N	Y
SP0036M47	PD		M	68	67		N							AL	N	W		N		Y	Y
DP438	PD		M	76	65	N	Y		Y	N	Y	4	FL	AL	N	W	N	Y	second	N	Y

SP0154	PD		F	74	70		Y	68						AL	N	W		Y	third+	N	Y
SP0165	PD		M	66	54		N							GA	N	W		N		Y	Y
DC226	NHC		M	67		N	N		N	N	N		LA	AL	N	W	N	N		N	N
DP064	PD		M	65	58	N	N		N	N	Y	29	MS	MS	N	W	N	Y	third+	N	N
DP151	PD	DC300	M	71	70	N	N		N	N	N		AL	AL	N	W	N	Y	second	N	N
DC170	NHC		M	77		N	N		N	N	N		AR	AL	N	W	N	N		Y	N
DP184	PD		F	69	58	N	N		N	N	Y	7	IN	AL	N	W	N	N		N	Y
SC0139	NHC		M	60			N		N	N	N				N	W	N	N		N	N
DP171	PD		M	69	65	N	Y	17	Y	N	N		OH	AL	N	W	N	N		Y	N
SC0141	NHC		F	69			N		N	N	N			AL		W	N	N		N	N
SC0582	NHC		F	58			N			N	N			AL	N	W	N	N		N	N
DC079	NHC	DP250	F	55		N	N			N	N		AL	FL	N	M	N	Y	third+		Y
DC156	NHC		F	56		N	N		N	N	N		AL	AL	N	W	N	N		N	N
DP446	PD		F	67	60	N	N			N			AL	AL	N	B	N	N		N	Y
SP1096	PD		M	70	69		N		N	Y	Y			MS	N	W	N	Y	second	N	Y
DP399	PD		F	67	50	N	N		N	N	Y but around or after onset	14	OH	AL	N	W	N	N		N	Y
SP0132	PD		F	72	60		N							AL	N	W		N		N	N
DP460	PD		M	72	66	N	N			N	N		AL	FL	N	W	N	N		Y	N
DP341	PD		M	80	70	N	Y	35	N	N	N		AL	AL	N	W	N	N		N	N
SP0039M42	PD		M	74	63		N							AL	N	W		N		N	N
SP0205	PD		F	85	69		N							AL	N	W		N		Y	Y
DC214	NHC		F	58		N	N		N	N	N		AL	AL	N	W	N	N		Y	N
DP570	PD	DC265	M	59	47.5	N	N		N	N	N		AL	AL	N	W	N	Y	second	N	N
DP271	PD		M	74	72	N	N		N	N	N		AL	AL	N	W	N	N		N	Y
DP431	PD		M	73	71	N	N		N	N	N		TX	AL	N	W	N	N		Y	Y
DP352	PD		M	62	51	N	Y after onset	52	N	N	Y	7		GA	N	W	N	Y	first	N	N
DC106	NHC	DP308	M	65		N	N		N	N	N		MS	MS	N	W	N	N		N	N
DP408	PD		M	23	17	Y	N		N	N	N		AL	AL	N	W	N	Y	first	Y	N
SC0111	NHC		F	70			N							GA	N	W		Y	second	N	N
DP585	PD		M	76	60	N	N		N	Y	Y	13	AL	AL	N	W	N	N		Y	Y
SP0151	PD		M	69	60		Y	20						AL	N	W		Y	first	N	N

SP0212	PD		M	83	72		N							AL	N	W		N		N	Y
DP199	PD		M	81	69	N	N		N		N		MS	AL	N	W	N	N		Y	Y
SC0628	NHC		F	63			N		N		N			AL	N	W	N	N		N	N
DP572	PD		F	61	60	N	N		N	N	N		MS	MS	N	W	N	Y	second	N	
DP117	PD		F	62	49	N	Y	13	N	N	Y	19	AL	AL	N	W	N	N		Y	Y
DP506	PD	DC160	F	78	73	N	N		N	N	Y	8		AL	N	W	N	Y	first	Y	Y
SC0039	NHC		F	66			N							FL	N	W		Y	first	N	N
SP0114	PD		M	80	64		N							AL	N	W		Y	first	Y	Y
DP201	PD		M	68	42	N	N		N	N	N		AL	AL	N	W	N	Y	first	N	Y
SP0228	PD		M	53	46		N							MS	N	W		N		Y	Y
DC078	NHC	DP249	M	78		N	N		N	N	Y	9	AL	AL	N	W	N	N		N	N
DP047	PD	DC022	M	74	69	N	N		Y	Y	N		AL	AL	N	W	Y			Y	N
DC279	NHC		F	58		N	N		N	N	N		GA	AL	N	W	N	N		N	N
DP626	PD	DC293	M	70	66	N	N		N	N	Y	17	IL	AL	N	W	N	Y	first	Y	Y
SC0075	NHC		F	56			N							AL	N	W		N		N	N
DP563	PD	DC261	F	80	77	N	N		N	N	N		AR	AL	N	W	N	Y	first	Y	Y
DP144	PD		M	66	42	N	Y	14	Y	N	N			AL	N	W				Y	N
SP1034	PD		M	51	36		Y	15	Y	N	Y			CO	N	W	N	N		N	N
DP278	PD		M	67	60	N	N			N	Y		AL	AL	N	W	N	N		N	N
SP0076	PD		M	71	65		Y	20						AL	N	B		N		N	
DP109	PD		M	74	57	N	Y	20	Y	N	N		GA	GA	Y	W	N	N		N	Y
DP272	PD		F	80	75	N	N		N	N	N		AL	AL	N	W	N	N		N	N
DP620	PD	DC290	M	70	62	N	Y	8	N	N	N		AL	AL	N	W	N	N		N	Y
SC0248	NHC		F	54			N		N	N	N			AL	N	W	N	N		N	N
SP1015	PD		M	87	82		N				N			AL	N	W	N	Y	first	N	N
DP241	PD		M	73	72	N	N			N	N		NJ	MS	N	W	N	N		N	Y
SC0121	NHC		F	72			N							GA	N	W		N		Y	N
DP566	PD		F	66	55	N	Y	3.5	N	N	N			AL	N	W				N	N
DC283	NHC		F	63		N	N			N	N		TN	TN	N	W	N	N		N	N
DP077	PD		F	53	44	N	N		N	N	N		AL	AL	N	W	N	N		N	
DP202	PD		M	69	66	N	N			N			GA	GA	N	W	N	N		N	N
DC162	NHC		F	73		N	N		N	N	N		NC	AL	N	W	N	N		N	N
DP402	PD	DC120	F	66	54	N	Y	18	N	N	N		NY	AL	N	W	N	Y	first	Y	Y

DP087	PD		M	60	54	N	N		N	N	Y	4	AL	AL	N	W	N	Y	first	N	N
DC161	NHC		F	73		N	N		N	N	N		Not US	AL	N	W	N	N		N	N
DP377	PD		M	63	62	Y	N		N	N	Y	12	LA	AL	N	W	N	N		N	Y
DP593	PD		M	58	47	N	N		N	N	N		MS	AL	N	W	N	N		N	N
DP513	PD		M	72	68	N	Y	18	N		Y	1	HI	AL	N	W	N	N		Y	N
DP270	PD		F	61	59	N	N		N	N	N		AL	AL	N	W	N	Y	second	N	N
SP1011	PD		M	60	43		N		N	N				AL	N	W	N	Y	second	N	Y
DP262	PD	DC087	F	61	57	N	N		N	N	Y		NY	FL	Y	W	N	Y	first	Y	Y
DP250	PD	DC079	M	59	53	N	Y	17	Y		N		AR	FL	N	W	N	N		N	N
SP0217	PD		M	70	59		N							AL	N	W		N		N	N
DC152	NHC		M	59		N	N		N	N	N		PA	AL	N	W	N	N		N	N
DP349	PD		F	54	46		N		N	N	N		AL	AL	N	W	N	N		Y	Y
DC117	NHC	DP385	M	82		N	N		N	N	N		AL	AL	N	W	N	N		N	N
DP581	PD	DC270	F	70	67	Y	N		N	N	Y	20	MS	MS	N	W	N	N		N	Y
DC060	NHC	DP169	F	70		N	N		N	N	Y	18	AL	AL	N	W	N	N		Y	N
DP554	PD		M	73	66	N	N				N		UT	AL	N	W	N	N		N	N
DP418	PD	DC127	F	66	55	N	N		N	N	Y	18	AL	AL	N	W	N	N		N	Y
SC0576	NHC		M	68			N		N		Y			AL	N	W	N	Y	second	N	N
SP0593	PD		M	55	53		N		N	N	N			AL	N	W	N	N		N	N
DC253	NHC		F	67		N	Y	5	N	N	N		TN	AL	N	W	N	N		N	N
DP414	PD		F	82	75	N	Y	4.5	N	N	N		AL	AL	N	W	N	N		N	N
DP590	PD		M	73	70	N	N		N	N	Y	4		AL	N	W	N	Y	first	N	N
DP131	PD		F	59	55	N	N		N	N	N		TX	AL	N	W	N	N		N	Y
DC076	NHC	DP245	F	77		N	N		N	N	N		AL	AL	N	W	N	Y	second	Y	Y
DP127	PD		M	72	66	N	N		N	N	N		TX	AL	N	W	N	N		N	Y
DC215	NHC		F	60		N	N		N	N	N		AL	AL		W	N	N		N	N
DC195	NHC		F	67		N	N		N	N	N		TN	AL	N	W	N	N		N	N
SC0020F43	NHC		F	73			N							AL	N	W		N		N	N
DC285	NHC	DP613	F	68		N	N		N	N	N		AL	AL	N	W	N	N		N	Y
SC0555	NHC		F	75			N		N	N	Y			AL	N	W	N	N		N	N
DC288	NHC		M	50		N	N		N	N	N		AL	AL	N	W		N		Y	N
SP0195	PD		M	75	58		N							AL	N	W		N		N	N
SP0969	PD		F	64	59		N		N	N	N			GA	N	W	N	Y	third+	N	N

DP103	PD		M	65	62	N	N		N	N	N		AL	AL	N	W	N	N		N	N
SP0964	PD		M	50	48		N		N	N	N			AL	N	W	N	Y	second	N	N
SP0152	PD		M	69	68		Y	19						AL	N	W		Y	third+	Y	N
DP474	PD		M	70	57	N	N		N	N	N		AL	AL	N	W	N	Y	second	Y	N
SP0958	PD		F	72	56		N		N	N	N			AL	N	W	N	N		Y	Y
SP0046M57	PD		M	58	55		Y	16						AL	N	W		Y	first	Y	N
SP0221	PD		M	52	44		N							AL	N	W		Y	first	N	N
DC041	NHC	DP095	M	75		N	N		N	N	N			AL						N	N
DP409	PD		F	68	64	N	Y	5.5	N	N	N		AL	AL	N	W	N	N		N	
SC0553	NHC		F	64			N		N	N	N			AL	N	W	N	N		N	N
DP476	PD		F	54	40	N	N		N	N	N		Not US	LA	N	W	N	N		N	Y
DP244	PD		M	65	64	N	N			N			AL	AL	N	W	N	N		Y	Y
SP0050M41	PD		M	75	74		N							AL		W		N		N	N
DC061	NHC	DP172	F	73		N	N		N	N	N		AL	AL		W	N	N		N	N
SP0095	PD		M	74	65		N							AL	N	W		N			
DP579	PD		M	68	67	N	Y	1	Y	N	Y	5	AL	AL	N	W		N		N	N
SC0636	NHC		F	76			N		N	N	N			AL	N	W	N	N		N	N
DP136	PD		F	57	54	N	N		N	N	N		GA	AL	N	W	N	N		N	N
DP344	PD		F	75	63	Y	N		N	N	N		AL	AL	N	W	N			Y	Y
SP1066	PD		F	66	59		Y	16	N	N	Y			AL	N	W	N	N		N	N
DP382	PD		F	74	64	N	N			N	Y	16	AL	AL	N	W	N	Y	first	N	N
SP0120	PD		M	66	51		N							AL	N	W		Y	first	N	N
SC0063	NHC		M	67			N							GA	N	W		N		N	N
DP110	PD		M	67	54	N	N		N	N	N		AL	AL	N	B	N	N		N	Y
DP357	PD		F	69	49	N	Y	23	N	N	N		AL	AL	N	W	N	N		N	N
DC245	NHC		F	49		N	N		N	N	N		AL	AL	N	B	N	Y	first	N	N
DP478	PD		M	76	66	N	N		N	N	N		MS	MS	N	W	N	N		Y	
DC155	NHC	DP501	F	60		N	N		N	N	N		AL	AL	N	W	N	N		Y	N
SC0151	NHC		F	58			N		N	N	N			AL	N	W	N	N		Y	Y
DC148	NHC		F	80		N	Y	64	N	N	Y	6	AL	AL	N	W	N	Y	first	N	N
DP061	PD		F	81	55	N	N		N	N	N		Not US	AL, Spain	N	W	N	N		N	N
SC0017F56	NHC		F	59			Y	45						AL	N	W		N		N	Y

SC0631	NHC		M	82			N		N	N	Y				N	W	N	Y	first	N	N
SP0187	PD		M	69	65		N							AL	N	W		N		N	N
DC307	NHC		F	61		N	N		N	N	N		CA	FL	N	W	N	N		N	N
DP458	PD		F	54	51	N	N		N	N	N		TN	AL	N	W	N	Y	second	N	N
SC0574	NHC		F	63			N		N	N	N				N	W	N	N		N	Y
SP0072	PD		M	78	60		N							AL	N	W		Y	first	N	Y
SP0724	PD		M	62	53		N			N	N			AL	N		Y	Y	second	N	N
DP430	PD		M	68	50	N	Y after onset	54	N	N	N		FL	AL		W	N	Y	first	N	N
DP166	PD		M	78	68	N	N			N	N		AL	AL	N	W	N	Y	first	N	N
DP229	PD		M	75	70	N	N				N		IN	AL	N	W	N	N		N	N
DP432	PD		F	73	64	N	Y	1.5	N		Y	18		AL	N	W	N	N		N	N
DP218	PD		M	84	81	N	Y	32	N	N	N			AL	N	W	N	N		Y	Y
DC112	NHC		F	61		N	Y	23	N	N	N		TX	AL	Y	W	N	N		N	N
DC168	NHC		M	70		N	N		Y	N	N		TX	AL	Y	AA	N	N		N	N
SP0955	PD		F	71	64		Y	19	N	N	N			AL	N	W	N	N		N	N
DP373	PD	DC125	M	65	56	N	Y		Y	N	N		AL	AL	N	W	N	Y	third+	Y	Y
SP0123	PD		M	66	55		N							AL	N	W		N		Y	Y
DC199	NHC	DP530	F	70		N	N			N	N		MS	MS	N	W	N	N		N	N
DP542	PD		M	67	42	N	Y	21	N	N	Y	3		AL	N	W	N	Y	first	N	Y
DP619	PD		M	58	38	N	N		N	N	N		AL	AL	N	W	N	Y	first		
DP556	PD		M	69	50	N	N		Y	N	Y	2	FL	AL	N	W	N	N		N	Y
DP178	PD		M	62	57	Y	Y	17	Y	N	Y	18	NC	MS	N	W	N	Y	second	N	N
DP423	PD		F	78	68	N	N		N	N	N		MS	MS	N	W	N			N	N
DP371	PD		F	66	32.5	N	N		N	N	Y	3	AL	AL	N	W	N	N		N	Y
SP0029M44	PD		M	71	69		N							AL	N	W		N		Y	Y
DC073	NHC		M	74		N	N			N	N		WA	AL	N	W	N	N		Y	N
SP1055	PD		M	46	30		N		N	N	N			AL	N	W	N	Y	third+	N	N
SP1076	PD		M	86	79		Y	79	N	N	N			AL	N	W	N	N		N	Y
SC0114	NHC		F	84			N							AL	N	W		N		N	N
SP0012	PD		F	60	58		N							AL	N	W		N		Y	N
DC020	NHC	DP042	F	71		N	N		N	N	N			AL		W				N	N
SP0075	PD		M	69	66		N		N	N	N			GA	N	W	Y	Y	second	Y	N
SP0116	PD		M	76	71		N							AL	N	W		N		N	Y

DP404	PD	DC123	M	74	64	N	Y	16	N	Y	N		AL	AL	N	W	N	N		N	Y
DP282	PD		M	84	70	N	N		N	N	N			AL	N	B				Y	N
SC0564	NHC		M	58			N		N	N	N			AL	N	W	N	N		N	N
SC0103	NHC		M	55			N							FL	N	W		Y	first	Y	
DC209	NHC		F	68		N	Y	14	N	N	N		AL	AL	N	W	N	N		N	N
DP020	PD	DC011	F	65	58	N	N			N	Y	2	AL	AL	N	W	N	Y	first	N	N
DC178	NHC		M	51		N	N		N	N	Y	19	TN	AL	N	W	N	N		N	N
SC0548	NHC		M	73			Y	57	N	N	N			NC	N	W	N	N		N	N
SC0137	NHC		F	61			N							AL	N	W		N		N	N
DP160	PD		M	81	73	N	N		N	Y	N		NC	FL	N	W	N	N		Y	N
DC103	NHC	DP304	M	73		N	Y	7	Y	N	N		TN	TN	N	W	N	N		N	N
DP444	PD		M	65	59	N	Y	16	Y	N	N		AL	AL	N	W	N	Y	third+	Y	N
DP424	PD		F	62	58	N	N		N	N	N		IL	AL	N	W	N	Y	second	N	N
SP0106	PD		M	65	55		N							AL	N	W		Y	third+	Y	Y
DP323	PD		F	75	65	N	N		N	N	N		PA	AL	N	W	N	N		Y	Y
DP532	PD	DC200	M	66	42	N	N		N	N	Y but around or after onset	10	IL	FL	N	W	N	N		N	Y
SC0586	NHC		F	63			N		N	N	N			AL	N	W	N	N		N	N
SP0260	PD		M	67	43		N		N	N	Y			Ms	N	W	N	N		N	Y
SP0064	PD		M	65	44		N							AL	N	W		N		N	N
DP574	PD	DC267	F	61	40	Y	N		N	N	N		AL	AL	N	W	N	N		Y	Y
DC037	NHC	DP073	F	61		N	N		N	N	Y	18	AL	AL	N	W	N	Y	second	N	N
DC127	NHC	DP418	M	79		N	N		N	N	N			AL	N	W	N	N		N	N
DP080	PD		F	56	54	N	Y	15	N	N	N		AL	AL	N	W	N	Y	second	N	N
SC0107	NHC		F	75			N							AL	N	W		N		N	N
SC0040	NHC		M	55			Y	18						AL	N	W		N		N	N
SP0048M45	PD		M	71	65		Y	7						AL	N	W		N		Y	N
DP383	PD		F	73	72	N	Y	20	N	N	N		FL	FL	N	W	N	Y	second	N	Y
SC0527	NHC		F	68			N		N	N	N			AL	N	W	N	Y	first	N	N
SP0979	PD		F	68	50		N		N	N	Y			MS	N	W	N	N		Y	Y
DP540	PD		M	68	60	N	N		N	N	N			AL	N	B	N	N		N	N
SP0944	PD		F	62	50		N		N	N	N				N	W	N	Y	second	Y	N

DP067	PD	DC033	M	66	48	N	N		N	N	Y		GA	AL	N	W	N	N		Y	Y
DP384	PD		M	70	66	N								AL	N	W				N	Y
SC0092	NHC		M	61			N							AL	N	W		N		N	N
SP0171	PD		F	64	59		N							AL	N	W		N		N	Y
SP0031M43	PD		M	72	67		N							AL	N	W		N		N	Y
DP644	PD		M	71	58	N	N			N	N		AL	AL	N	W	N	N		N	Y
SC0469	NHC		M	62			N		N	N	N			AL	N	W		N		N	N
DP604	PD		M	65	62	N	N		N	N	N		TN	AL	N	W	N	N		N	Y
DC165	NHC		F	71		N			N	N	N		AL	AL	N	W	N	Y	first	N	N
SP1051	PD		M	70	67		N		Y	N	N			AL	N	W	N	N		N	N
DC036	NHC		F	68		N	N		N	N	Y	17	AL	AL	N	W	N	N		N	N
DP511	PD		M	63	61	N	N		N	N	N		AL	AL	N	W	N	N		N	N
DC219	NHC		F	58										AL	N	W					
DC296	NHC		F	45		N	N		N	N	Y	14	NE	AL	N	W	N	N		N	N
DP208	PD	DC070	M	62	55	N	N		N	N	N		WV	AL	N	W	N	N		N	N
SP1093	PD		M	70	65		N		N	N	N			FL	N	W	N	Y	second	N	N
DC150	NHC		F	55		N	N			N	N		AL	AL	N	B	N	Y	first	Y	Y
SP1021	PD		M	54	50		N		N	N	N			AL	N	W	N	N		N	N
DP167	PD		F	82	75	N	N		N	N	N		SC	AL	N	W	N	N		N	Y
DP309	PD	DC107	M	55	49	N	N			N	Y	42	AL	AL	N	W	N	N		N	Y
DC013	NHC	DP022	F	65		N	N		N	N	N		AL	AL	N	W	N	Y	second	N	Y
SP0098	PD		M	65	51		N							AL	N	W		N			N
SP0277	PD		M	68	67		Y	23	N	N	N			AL	N	W	N	N		N	N
SP1002	PD		F	51	46		N		N	N	N			WA	N	W	N	N		N	Y
DP328	PD		M	67	61	N	Y	4.5	Y	N	N		NY	AL	N	W	N	N		N	N
SP0268	PD		M	61	55		N		N	N	Y			AL	N	W	N	Y	second	N	N
SP0256	PD		M	70	64		N		N	N	N			Al		W	N	N		Y	Y
DP294	PD		M	72	60	N	N		N	N	Y	21	AL	AL	N	W	N	Y	first	N	N
DC029	NHC	DP060	F	35		N	N		N	N	Y	9	AL	AL	N	W	N	N		Y	N
SC0002	NHC		M	76			N							FL	N	W		Y	first	N	N
SC0109	NHC		M	85			Y	71						AL	N	W		N		N	N
DP192	PD	DC066	M	84	72	N	Y	18	N	N	N		AL	AL	N	W	N	Y	first	N	Y
DP132	PD		F	69	60	N	N		N	N	N		MS	MS	N	W	N	N		N	N

DP622	PD		F	77	70	N	N		N	N	Y	3	AL	AL	N	W	N	Y	first	N	Y
SC0093	NHC		F	70			N							AL	N	W		N		N	N
DC154	NHC	DP492	F	66		N	N		N	N	N		AL	AL	N	W	N	N		N	N
DP520	PD		M	71	60	N	N		N	N	N		AL	AL	N	W	N	Y	first	Y	N
DC050	NHC		F	88		N	N		N	N	N		AL	AL	N	W	N	N		N	N
SP0014	PD		F	68	40		N							AL	N	W		N		N	N
DP104	PD		M	82	81	N	Y	14	N	N			NC	AL	N	W	N	N		N	Y
DC254	NHC	DP646	F	65		N	N		N	N	N		KY	AL	N	W	N	N		N	Y
SC0623	NHC		F	57			N		N	N	N			AL	N	W	N	N		N	N
DP083	PD		F	72	60	N	N		N	N	N		AL	AL	N	W	N	N		Y	Y
SC0071	NHC		M	66			Y	13						AL	N	W		N		N	N
DP628	PD		M	66	50	N	Y	15	Y	N	Y	65	AL	AL	N	W	N	N		Y	N
SP0197	PD		F	53	13		Y after onset	16						FL	N	W		N		Y	N
SP0983	PD		M	58	43		N		N	N	Y			MS	N	W	N	N		Y	Y
DC308	NHC		F	61		N	N		N	N	N		AL	AL	N	W	N	Y	first	N	N
DP552	PD		F	75	71	N	N		N	N	N		AL	AL	N	W	N	Y	first	N	Y
SC0024F44	NHC		F	71			N							AL	N	W		N		N	
SP0089	PD		M	78	69		N							AL	N	W		Y	first	N	N
DC261	NHC	DP563	M	81		N	N		N	N	N		TX	AL	N	W	N	N		N	N
SC0630	NHC		F	65			N		N	N	N			FL	N	W	N	N		N	N
DC206	NHC		M	71		N	N		N	N	N		FL	FL	N	W	N	N		N	N
SP1050	PD		M	71	61		N		N	N	Y			MS	N	W	N	Y	third+	N	Y
DP459	PD		F	66	62	N	N		N	N	N		AL	AL	N	W	N	Y	third+	N	N
SP0006	PD		M	72	71		Y	12						AL	N	W		Y	first	N	Y
SC0149	NHC		M	65			Y	65	N	N	N			AL	N	W	N	N		N	N
DP603	PD		M	75	74	N	N		N	N	N		AL	AL	N	W	N	N		N	Y
DP611	PD		F	73	64	N	Y	18	N		Y	22	MS	MS	N	W	N	Y	second	Y	Y
DC119	NHC	DP401	F	70		N	N		N	N	Y	70		AL	N	W	N	N		N	Y
DP279	PD	DC094	M	71	58	N	N		N	Y	Y	3	AL	MS	N	W	N	N		N	
DC259	NHC		M	64		N	Y	8	N	N	N		AL	AL	N	W	N	N		N	N
DC179	NHC		F	56		N	N		N	N	N		AL	AL	N	W	N	N		N	N
DC069	NHC	DP207	M	64		N	Y	23	Y	N	N		LA	MS	N	W	N	N		Y	N
SP0134	PD		F	60	54		N							AL	N	W		Y	first	N	N

DP123	PD		M	76	60	N	N		N	Y	Y	26	AL	AL	N	W	N	N		N	N
DP359	PD		F	69	67	N	N		N	N	N		AL	AL	N	W	N	N		N	N
DC302	NHC		M	64		N	N		N	N	Y	6	IN	AL	N	M	N	N		N	N
SC0183	NHC		F	64			N		N	N	N			MS	N	W	N	N		Y	N
DC294	NHC	DP632	M	70		N	N		N	N	N		GA	GA	N	W	N	N		N	N
DC196	NHC	DP525	M	78		N	N			N	Y	18	FL	AL	N	W	N	N		N	N
SP0059	PD		M	87	80		N							AL	N	W		N		N	Y
DC052	NHC		M	74		N	N			N			AL	AL	N	W	N	N		N	N
DP495	PD		M	55	52	N	N		N	N	N		AL	AL	N	B	N	N		N	N
SP0070	PD		M	75	68		N							TN	N	W		N		Y	Y
DP297	PD		M	78	63	N	N		N	N	N		AL	AL	N	W	N	Y	first	Y	Y
DP158	PD		M	79	68	Y	Y	42	N		N			AL	N	W				N	N
SP0954	PD		M	72	70		N		Y	Y	N			FL	N	W	N	N		Y	N
DC063	NHC	DP186	F	76		N	N			N	N		AL	AL	N	W	N	N		N	
DP434	PD		F	59	48									AL		B					
DC158	NHC		F	64		N	N		N	N	N		AL	AL	N	W	N	Y	first	Y	N
SC0634	NHC		F	63			N		N	N	N			AL	N	W	N	N		Y	N
DC169	NHC		F	68		N	N		N	N	N		TX	AL	N	W	N	Y	first	N	Y
DP220	PD		M	74	61	N	N		N	N	N		AL	AL	N	W	N	N		N	N
SC0066	NHC		M	63			N							AL	N	W		N		N	N
SP0110	PD		M	66	62		Y	14						AL	N	W		Y	second	Y	Y
DP019	PD		M	54	39	N	N		N	N	N		AL	AL	N	W	N	N		N	N
DP180	PD		M	68	55	N	N		N	N	Y	3	TN	TN	N	W				Y	N
DC213	NHC		M	75		N	N		N	N	N		FL	MS	N	W	N	N		N	N
DC024	NHC	DP050	F	53		N	N		N	N	Y	11	CA	AL	N	W	N	N		Y	Y
DP011	PD		M	73	65	N	N		N	N	Y	10	IL	AL	N	W	N			N	N
DP111	PD		M	69	64	N	N		N	N	N		AL	AL	N	W	N			N	Y
DC171	NHC	DP514	F	60		N	N		N	N	N		FL	AL	N	W	N	N		N	N
DP026	PD	DC016	F	68	64	N	N		N	N	N		AL	AL	N	W	N	Y	second	Y	N
SP0267	PD		M	79	58		Y	57	Y	N	N			Al	N	W	N	Y	third+	N	N
SP0158	PD		F	75	68		N							MS	N	W		Y	first	Y	Y
DC232	NHC		M	48		N	N		Y	N	N		OK	AL	N	W	N	Y	first	N	N
SC0049	NHC		F	62			Y	9						AL	N	W		N			Y

SP1109	PD		M	78	75		Y	72	N	N	Y			AL	N	W	N	N		N	N
SC0619	NHC		M	68			N		N	N	N			AL	N	W	N	N		N	N
DP195	PD		M	74	71	Y	N		N	N	Y		FL	FL	N	W	N	N		N	N
DC015	NHC	DP025	F	63		N	N		N	N	Y	63	AL	AL	N	W	N	N		N	N
DC033	NHC	DP067	F	66		N	N			N	N		AL	AL	N	W	N	N		N	
SP0037M59	PD		M	56	33		N							AL	N	W		N		N	Y
SC0144	NHC		M	66			N		Y	N	Y			AL	N	W	N	N		N	N
DP057	PD		M	76	75	N	N		N	N	N		IL	AL	N	M	N	Y	first	N	N
SP0004	PD		F	71	66		N							FL	N	W		N		Y	N
DP353	PD	DC114	F	59	54	N	N		N	N	Y	18	AL	AL	N	W	N	N		N	Y
SC0117	NHC		F	62			N							AL		W		N		N	N
DP631	PD		M	73	65	N	N		N	Y	N		AR	AL	N	W	N	Y	first	N	N
DP491	PD	DC153	M	76	74	N	N		N	Y	Y	30	AR	AL	N	W	N	N		N	N
DP069	PD		M	76	66	N	N		Y	N	N		MS	MS	N	W	N	N		N	Y
DP081	PD		F	68	55	N	N		N	N	N		AL	AL	N	W	N	N		N	N
DC305	NHC		F	66		N	N		N	N	N		AL	AL	N	W	N	N		N	N
SP0978	PD		M	65	61		Y	9	Y	N	Y			AL	N	W	N			Y	N
DC183	NHC		M	70		N	N		N	N	N		AL	AL	N	W	N	N		N	N
SC0083	NHC		M	65			N							AL	N	W		N		N	N
SP0083	PD		M	72	64		Y	11						AL	N	W		Y	first	Y	Y
DP161	PD		M	65	56	N	N		N	N	N			AL	N	W				N	N
DP337	PD		F	68	64	N	N		N	N	Y	45	MS	MS	N	W	N	N		N	Y
DP264	PD		F	67	63.5	N	N		N	N	N		GA	AL	N	W	N	Y	first	N	N
DP246	PD		F	80	79	N	Y	77	N	N			AL	AL	N	W	N	N		N	N
SP0210	PD		M	77	68		N							AL	N	W		N		N	Y
SP0087	PD		F	71	68		N							AL	N	W		N		N	N
DP524	PD		F	81	79	N	N		N	N	Y	2		AL	N	W				N	Y
DC011	NHC	DP020	M	67		N	N		N	N	Y	2	AL	AL	N	W	N	Y	second	N	N
DP326	PD		M	63	43.5	Y	N		N	N	N		AL	AL	N	W	N			Y	N
DC135	NHC		M	66		N	N		N	N	N		AL	AL	N		N	N		N	N
DP260	PD		M	64	57	N	N		N	N			AL	AL	N	W	N	Y	second	Y	Y
SC0596	NHC		F	68			N		N	N	N			AL	N	W	N	N		N	N
DP650	PD		F	66	59		N		N	N	N			MS	N	W	N	Y	first	Y	N

SC0123	NHC		F	53			N							MS	N	W		N		N	N
DP397	PD		F	60	43	N	N		N	N	N		MD	AL	N	W	N	N		N	N
SP0167	PD		M	74	72		N							AL	N	W		N		N	N
DP441	PD		M	77	73	N	Y	14	N	N	N		WV	AL	N	W	N	Y	first	N	N
DP130	PD		M	70	62		N		N	N	Y	2	AL	AL	N	W	N	N		N	N
SC0061	NHC		F	61			N							AL	N	AA		N		N	N
SC0534	NHC		F	55			N		N	N	N			AL	N	W	N	Y	second	N	Y
DP187	PD		M	63	57	N	N			N	N		FL	AL	N	W	N	N		N	
DC164	NHC		F	53		N	Y	49	N	N	N		Not US	AL	N	W	N	N		N	N
DC246	NHC		F	70		N	N		N	N	N		AL	AL	N	B	N	N		N	N
DC277	NHC		F	63		N	N		N	N	N		MS	AL	N	W	N	Y	third+	N	N
DC293	NHC	DP626	F	72		N	N		N	N	Y	12	AL	AL	N	M	N	N		Y	N
DC273	NHC		F	68		N	N		N	N	N		WV	AL	N	W	Y	Y	second	N	N
SP0223	PD		M	61	40		N							AL	N	W		Y	first	N	N
DP498	PD		M	81	78	N	N		N	Y	N		ID	AL	N	W	N	N		Y	Y
DC136	NHC		F	58		N	N		N	N	N		NE	AL	N	W	N	N		N	N
DP317	PD		M	75	58	N	N		N	N	N		MS	MS	N					N	Y
SP1003	PD		M	58	51		N		N	N	N			AL	N	W	N	Y	second	Y	Y
SP0184	PD		M	80	70		N							FL	N	W		Y	first	Y	Y
DP224	PD		F	73	59		Y	35	N	N	Y			SC	N	W				Y	Y
SC0610	NHC		F	60			N		N	N	N			AL	N	W	N	Y	second	N	N
DP327	PD		M	64	62	N	N		N	N	N		GA	GA	N	W	N	N		N	Y
SP0616	PD		M	50	48		N		N	N	N			AL	N	W	N	N		Y	N
SP0044M58	PD		M	57	43		N							AL	N	W		N		N	Y
DP525	PD	DC196	F	75	70	N	N		N	N	Y	16	AL	AL	N	W	N	Y	first		Y
DC057	NHC	DP138	F	56		N	N		N	N	N		AL	AL	N	W	N	N		Y	N
DP538	PD		F	59	55	N	Y	7	N	N	N		FL	AL	N	W	N	Y	second	N	N
DC101	NHC		F	62		N	N		N	N	Y	7	AL	AL	N	W	N	N		N	Y
SC0138	NHC		M	62			Y	22	N	N	N			OR	N	W	N	N		N	N
DP595	PD		M	63	60	N	N		N	N	N		AL	AL	N	W	N	N		Y	N
SC0621	NHC		F	57			N		N	N	Y			AL	N	W	N	Y	third+	N	N
DP448	PD	DC140	M	62	57	N	Y	14	N	N	N		AL	AL	N	W	N	Y	first	Y	Y
SP0173	PD		M	56	31		Y	30						AL	N			Y	second	N	Y

DC138	NHC	DP437	F	70		N	N			N	N		Not US	AL	N	W	N	N		N	N
DP035	PD		F	50	48	N	N		N	N	N		AL	TN	N	W	N	N		N	N
DP606	PD		F	64	54	N	N		N	N	N		AL	AL	N	W	N	Y	third+	N	N
SP0055	PD		M	72	62		N							AL	N	W		N		N	N
DP642	PD		F	71	69	N	Y	67	N	N	N		AL	AL	N	W	N	N		N	N
DP597	PD		M	78	75	N	N		N	N	Y		AL	AL	N	W	N			N	N
SC0622	NHC		F	78			N		N	N	N			AL	N	W	N	N		Y	N
DP245	PD	DC076	M	77	70	N	N		N	N	N		AL	AL	N	W	N	N		Y	Y
SP0139	PD		F	65	62		N							MS	N	W		N		N	Y
DP485	PD		M	65	58		N		Y	N	N		AL	AL	N	W	N	N		Y	Y
DP507	PD		M	60	35	N	Y		Y		Y	3	IN	AL	N	W	N	N		N	Y
DP469	PD		F	69	59	N	N		N	N	N		AL	AL	N	W	N	N		N	Y
DP065	PD	DC032	M	64	61	N	N		Y	N	N		AL	AL	N	W	N	N		N	N
SP0209	PD		M	63	57		N							AL	N	W		N		N	N
SP1056	PD		M	66	60		Y	15	Y	Y	Y			AL	N	W	N	N		N	Y
SP0278	PD		M	55	38		N		N	N	N			AL	N	W	N	Y	first		
DC102	NHC	DP300	F	67		N	N		N	N	Y	15	TN	AL	N	W	N	N		N	N
DC218	NHC		F	62		N	Y	12		N	N		GA	AL	N	W	N	N		N	N
SP0185	PD		F	63	55		N							AL	N	W		N		N	N
SC0060	NHC		F	73			N							MS	N	W		N		N	Y
DP025	PD	DC015	M	65	50	N	Y	13	Y		Y	65	AL	AL	N	W	N	N		N	Y
SP0078	PD		F	66	66		Y	8						MS	N	W		Y	second	N	N
SC0105	NHC		F	76			N							AL	N	W		N		N	N
SC0605	NHC		F	57			N		N	N	N			AL	N	W	N	N		N	N
SP0977	PD		F	81	68		N		N	N	Y			AL	N	W	N			N	Y
DC072	NHC	DP223	M	83		N	N		N	N	N		CA	AL	N	W	N	N		N	N
SC0001	NHC		F	50			N							AL	N	W				N	N
DC065	NHC	DP189	F	69		N	N		N	N	N		AL	AL	N	W	N	N		N	N
DC019	NHC	DP032	F	65		N	N		N	N	N		LA	AL	N	W	N	Y	first	N	N

Supplementary Material / Questions

Question	Response options
Sex	❒ M ❒ F
Today's date (*as subject is completing form*)	____________________
Date of birth	____________________
Do you have Parkinson's disease?	❒ No ❒ Yes
How old were you when you first noticed a sign of Parkinson's disease (age at onset)?	____________________ years old
Do you have rapid eye movement sleep behavior disorder (RBD)?	❒ No ❒ Yes
Have you had a stroke, ataxia, multiple sclerosis, Alzheimer's disease, dementia, dystonia, autism, bipolar disorder, amyotrophic lateral sclerosis (ALS), or epilepsy?	❒ No ❒ Yes
Are you often constipated (fewer than 3 bowel movements per week occurring frequently)?	❒ No ❒ Yes
Have you lost more than 10 pounds in the last year?	❒ No ❒ Yes
Have you had a head injury that caused loss of consciousness or required medical care?	❒ No ❒ Yes
If yes, how old were you when it first happened	____________________ years old
Did you have repeated blows to the head such as in sports or military?	❒ No ❒ Yes
Were you ever exposed to Agent Orange or other chemical warfare?	❒ No ❒ Yes ❒Don't know
Were you ever exposed to heavy uses of pesticides or herbicides, for example, did you live on or near farms that did crop dusting?	❒ No ❒ Yes
If yes, how old were you (give the range, for example, from birth until age 16)	____________________
Are you Hispanic or Latino?	❒ No ❒ Yes
What race do you most identify yourself with?	❒White<br>❒Black or African American<br>❒American Indian/Alaskan Native<br>❒Asian<br>❒Native Hawaiian or other Pacific Islander<br>❒More than one race
Are you of Jewish ancestry?	❒ No ❒ Yes
Do you have any blood relatives who have Parkinson's disease (including your parents, grand-parents, siblings, aunts, and uncles)?	❒ No ❒ Yes
If yes, for each relative who has or had PD, list their relationship to you	____________________

These were exact questions used to collect the data that were used in this study. They were parts of Environmental and Family History Questionnaire. Age was calculated by subtracting date of birth from today's date. Positive family history was defined as having at least one first or second degree relative with PD. Question on having PD was mainly for QC purposes; PD diagnosis was made by one of our movement disorder specialist neurologists at University of Alabama at Birmingham. Question on neurological diseases (stroke, ataxia, etc.) was the exclusion criteria for controls. The questions on repeated blows to the head, exposure to heavy uses of pesticide/herbicide, and Agent Orange/chemical warfare were added midway in the first wave of data collection. The question on RBD, and duration of exposure to pesticides/herbicides were added at the start of second wave. The numbers of subjects for each question or combinations can be extracted from the dataset which is provided in **Supplemental Material / Data**.

Rationale on the construction of questions: Questions were intentially made simple to ensure subjects would understand and respond accurately (per IRB, 8th grade level education). Brevity was also important to retain subject's focus and interest. The rationale for herbicide/pesticide question was to gage exposure to toxicants that are used at home, work and agricultural setting. We also asked for age at exposure and duration to place it in relation to onset of PD. We collected data on occupation as well, but it was less informative than the direct simple question of "have you been exposed to herbicides/pesticides".

For the military-related chemicals, the main concerns were chemical warfare agents, and heavy uses of herbicides in Vietnam which were commonly referred to as Agent Orange.

Supplementary Material / Race and spousal relationship.

This supplement addresses and rules out potential confounding by race/ethnicity, and whether spousal pairs of PD-NHC differ from non-spousal PD and NHC singletons. The following tables show that having excluded non-Hispanic and non-White subjects, spousal and non-spousal subjects, shown side by side and bolded, are similar in (A) their characteristics and features of PD, (B) association of risk factors with PD in univariable and (C) multivariable analyses, (D) and population attributable fraction estimates.

Race, Ethnicity: To assess potential confounding by race/ethnicity, using **Supplementary Material /Data** of 1223 individuals**,** we excluded subjects who reported in Race as Asian, American Indian/ Alaskan Native, Black or African American, or More than one race, and those who reported as Hispanic for Ethnicity. Their sample sizes were too small for separate analysis. We excluded an additional 4 subjects who did not report on race nor ethnicity. We retained 26 subjects who reported as White and did not answer the Hispanic question because we know from asking the subjects that once they check White, many think the question on Hispanic is redundant (i.e., if White, then it is not Hispanic). We also retained 4 subjects who did not answer race but checked non-Hispanic, because, given that 96% of cohort is White, the 4 impute to 3.8 being White. This resulted in exclusion of 63 subjects, and a remaining sample size of 1160.

Spousal and non-spousal: Among the 1223 individuals in The **Supplementary Material /Data,** 116 PD and 116 NHC were spousal pairs, and 692 PD and 299 NHC were singletons**.** After exclusion for race and ethnicity, within the 1160 remaining subjects, 106 PD and 106 NHC were spousal pairs, and 657 PD and 291 NHC were singletons.

(A) Enrollment, subject characteristics, and features of PD in non-Hispanic Whites

	**Spousal pairs**						**Non-Spousal singletons**					
	**PD**		**NHC**		**PD features**		**PD**		**NHC**		**PD features**	
**Subject characteristics**	**N with data**	**Summary statistics**	**N with data**	**Summary statistics**	**OR [95%CI]**	**P**	**N with data**	**Summary statistics**	**N with data**	**Summary statistics**	**OR [95%CI]**	**P**
Sample size	106	-	106	-	-	-	657	-	291	-	-	-
Age	106	69·3±7·8	106	68·0±8·1	-	-	657	68·4±8·8	291	65·5±8·8	-	-
PD age at onset	106	60·2±9·9	-	-	-	-	657	59·4±11·2	-	-	-	-
Jewish ancestry	105	1 (1·0%)	106	1 (1·0%)	-	-	458	15 (3·3%)	198	8 (4·0%)	-	-
Residents of Deep South	106	103 (97%)	106	103 (97%)	-	-	649	618 (95%)	279	256 (92%)	-	-
Residents of DS Periphery	106	3 (2·8%)	106	3 (2·8%)	-	-	649	28 (4·3%)	279	22 (7·9%)	-	-
**PD Features**												
Sex (N & % male)	106	75 (71%)	106	31 (29%)	-	3E-09	657	408 (62%)	291	87 (30%)	-	1E-19
Constipation	102	53 (52%)	101	16 (16%)	**5·6 [2·9-10·8]**	1E-07	633	275 (43%)	284	37 (13%)	**5·1 [3·5-7·4]**	5E-19
RBD	104	11 (11%)	104	0 (0%)	**25·7 [1·5-442·2]**	2E-03	341	31 (9%)	109	0 (0%)	**22·2 [1·3-366·2]**	2E-03
Weight loss	104	29 (28%)	104	17 (16%)	**2·0 [1·0-3·8]**	6E-02	642	170 (26%)	285	37 (13%)	**2·4 [1·6-3·5]**	8E-06

(B) Association of individual risk factors with PD in non-Hispanic Whites

		Spousal pairs						Non-spousal singletons					
		Summary statistic		(a) Univariable analysis testing each variable individually				Summary statistic		(a) Univariable analysis testing each variable individually			
		PD	NHC	N with data		PD vs. NHC		PD	NHC	N with data		PD vs. NHC	
				PD	NHC	OR [95%CI LB]	P			PD	NHC	OR [95%CI LB]	P
**Males**	Age	69·9±7·1	70·8±9·3	75	31	0·99 [0·94]	0·69	68·8±8·8	67·1±9·2	408	87	1·02 [1·00]	5E-02
	Family history of PD	22 (30%)	2 (7%)	73	30	**6·04 [1·69]**	1E-02	119 (31%)	9 (10%)	380	86	**3·90 [2·12]**	1E-04
	MTBI/concussion	19 (26%)	4 (13%)	73	31	2·37 [0·89]	7E-02	85 (22%)	16 (18%)	393	87	1·23 [0·74]	0·25
	MTBI/concussion >10 yrs. prior to PD onset	18 (25%)	4 (13%)	73	31	2·21 [0·82]	9E-02	72 (18%)	16 (18%)	393	87	0·99 [0·60]	0·51
	Repeated blows to head	13 (19%)	2 (7%)	69	27	**2·90 [0·78]**	9E-02	57 (22%)	7 (11%)	262	62	**2·18 [1·08]**	3E-02
	Pesticide/herbicide exposure	21 (29%)	6 (20%)	72	30	**1·65 [0·69]**	0·17	85 (30%)	8 (12%)	278	65	**3·14 [1·63]**	2E-03
	Pesticide/herbicide exposure duration, yrs.	6·0±15·1	5·6±15·3	65	30	1·00 [0·98]	0·31	4·7±12·3	1·0±4·0	187	30	1·07 [0·99]	7E-02
	Military-related chemical exposures	8 (12%)	2 (7%)	69	30	**1·84 [0·47]**	0·23	17 (6%)	0 (0%)	279	64	**8·60 [1·46]***	2E-02
**Females**	Age	67·7±9·2	66·9±7·3	31	75	1·03 [1·01]	0·30	67·7±8·8	64·8±8·5	249	204	1·03 [1·02]	3E-04
	Family history of PD	19 (63%)	14 (19%)	30	72	**2·85 [2·04]**	2E-05	78 (34%)	32 (16%)	228	201	**2·75 [1·86]**	1E-05
	MTBI/concussion	4 (14%)	5 (7%)	28	74	1·97 [1·27]	0·12	40 (17%)	18 (9%)	241	201	2·02 [1·23]	9E-03
	MTBI/concussion >10 years prior to PD onset	4 (14%)	5 (7%)	28	74	1·56 [1·00]	0·12	32 (13%)	18 (9%)	241	201	1·56 [0·93]	7E-02
	Repeated blows to head	0 (0%)	0 (0%)	29	63	NT	NT	0 (0%)	0 (0%)	174	124	NT	NT
	Pesticide/herbicide exposure	11 (38%)	13 (18%)	29	72	**3·26 [2·19]**	2E-02	62 (35%)	16 (12%)	179	132	**3·84 [2·31]**	7E-06
	Pesticide/herbicide exposure duration, yrs.	6·7±13·9	5·0±15·0	28	71	1·02 [1·01]	0·30	6·1±13·5	1·0±3·3	125	78	1·11 [1·05]	1E-03
	Military-related chemical exposures	0 (0%)	0 (0%)	30	73	NT	NT	0 (0%)	0 (0%)	182	132	NT	NT

*Firth's penalized logistic regression (logistf R package v 1.26) was used for this test because the count for NHC males exposed to military-related chemicals is zero.

(C) Association of each risk factors with PD adjusted for other risk factors in non-Hispanic Whites

		Spousal pairs				Non-spousal singletons			
		(b) Multivariable analysis testing each variable adjusted on others				(b) Multivariable analysis testing each variable adjusted on others			
		N with data		PD vs. NHC		N with data		PD vs. NHC	
		PD	NHC	Adj OR [95%CI LB]	P	PD	NHC	Adj OR [95%CI LB]	P
**Males**	Age	58	26	0·99 [0·94]	0·59	220	58	1·04 [1·01]*	1E-02
	Family history of PD			**5·61 [1·48]**	2E-02			**4·54 [2·08]***	2E-04
	MTBI/concussion			2·04 [0·63]	0·16			0·78 [0·41]*	0·27
	MTBI/concussion >10 yrs. prior to PD onset			NT	NT			NT	NT
	Repeated blows to head			**2·69 [0·63]**	0·13			**2·02 [0·95]***	6E-02
	Pesticide/herbicide exposure			**2·11 [0·64]**	0·15			**2·80 [1·39]***	6E-03
	Pesticide/herbicide exposure duration, yrs.			NT	NT			NT	NT
	Military-related chemical exposures			**3·07 [0·46]**	0·16			**6·39 [1·03]***	<5E-02
**Females**	Age	25	69	0·99 [0·94]	0·60	159	129	1·04 [1·02]	1E-03
	Family history of PD			**7·37 [2·99]**	1E-04			**2·57 [1·56]**	1E-05
	MTBI/concussion			2·32 [0·64]	0·14			1·18 [0·62]	0·33
	MTBI/concussion >10 years prior to PD onset			NT	NT			NT	NT
	Repeated blows to head			NT	NT			NT	NT
	Pesticide/herbicide exposure			**3·24 [1·28]**	2E-02			**3·33 [1·94]**	1E-04
	Pesticide/herbicide exposure duration, yrs.			NT	NT			NT	NT
	Military-related chemical exposures			NT	NT			NT	NT

*Firth's penalized logistic regression (logistf R package v 1.26) was used for this test because the count for NHC males exposed to military-related chemicals is zero.

(D) Population attributable fraction in non-Hispanic Whites

		Spousal pairs			Non-spousal pairs		
		(c) Population attributable fraction for modifiable risks			(c) Population attributable fraction for modifiable risks		
		Determinants of PAF		**PAF (95% CI)**	Determinants of PAF		**PAF (95% CI)**
		Adj OR	Prev. in PD		Adj OR	Prev. in PD	
Males	Repeated blows to head	2·69	15%	**10% [-8%-27%]**	2·02*	20%	**10% [-0·4%-22%]**
Males	Pesticide/herbicide exposure	2·11	22%	**12% [-10%-34%]**	2·80*	30%	**20% [8%-31%]**
Males	Military-related chemical exposures	3·07	9%	**6% [-10%-21%]**	6·39*	7%	**6% [1%-13%]**
Males	Joint PAF	-	41%	**25% [-31%-62%]**	-	44%	**32% [9%-54%]**
Females	Pesticide/herbicide exposure	3·24	35%	**30% [0·6%-60%]**	3·33	34%	**24% [12%-35%]**

*Adjusted odds ratio taken from Firth's penalized logistic regression as listed in table C.

Supplementary Material / Software

Software or database name	Version	URL	RRIDs if applicable
PROGENY	9	http://www.progenygenetics.com/	RRID:SCR_006647
Microsoft Excel	16.0.15601.20526	https://www.microsoft.com/en-gb/	RRID:SCR_016137
R	4.1.3	https://cran.r-project.org/bin/windows/base/old/4.1.3/	RRID:SCR_001905
RStudio Connect	2023.03.0	https://docs.posit.co/previous-versions/connect/	RRID:SCR_000432
AF R Package	0.1.5	https://cran.r-project.org/package=AF	
data.table R Package	1.14.0	https://cran.r-project.org/package=data	
ggplot2 R Package	3.4.2	https://cran.r-project.org/package=ggplot2	
maps R Package	3.4.1	https://cran.r-project.org/package=maps	
mapsproj R Package	1.2.11	https://cran.r-project.org/package=mapsproj	
officer R Package	0.6.1	https://cran.r-project.org/package=officer	
openxlsx R Package	4.2.3	https://cran.r-project.org/package=openxlsx	
pairwiseCI R Package	0.1-27	https://cran.r-project.org/package=pairwiseCI	
renv R Package	0.13.2	https://cran.r-project.org/package=renv	
table1 R Package	1.4.3	https://cran.r-project.org/package=table	
targets R Package	0.13.1	https://cran.r-project.org/package=targets	
logistf R package	1.26	https://cran.r-project.org/package=logistf	
